# Genome-wide analysis of polyamine biosynthesis genes in wheat reveals gene expression specificity and involvement of STRE and MYB-elements in regulating polyamines under drought

**DOI:** 10.1186/s12864-022-08946-2

**Published:** 2022-10-30

**Authors:** Heba Talat Ebeed

**Affiliations:** Botany and Microbiology Department, Faculty of Science, Damietta University, Damietta, 34517 Egypt

**Keywords:** *Cis*-elements, Drought, Gene expression, Genome-wide analysis, Polyamines, Subcellular localization

## Abstract

**Background:**

Polyamines (PAs) are considered promising biostimulants that have diverse key roles during growth and stress responses in plants. Nevertheless, the molecular basis of these roles by PAs has not been completely realized even now, and unfortunately, the transcriptional analyses of the biosynthesis pathway in various wheat tissues have not been investigated under normal or stress conditions. In this research, the findings of genome-wide analyses of genes implicated in the PAs biosynthesis in wheat (*ADC*, Arginine decarboxylase; *ODC*, ornithine decarboxylase; *AIH*, agmatine iminohydrolase; *NPL1*, Nitrlase like protein 1; *SAMDC,* S-adenosylmethionine decarboxylase; *SPDS*, spermidine synthase; *SPMS,* spermine synthase and *ACL5,* thermospermine synthase) are shown.

**Results:**

In total, thirty PAs biosynthesis genes were identified. Analysis of gene structure, subcellular compartmentation and *promoters* were discussed. Furthermore, experimental gene expression analyses in roots, shoot axis, leaves, and spike tissues were investigated in adult wheat plants under control and drought conditions. Results revealed structural similarity within each gene family and revealed the identity of two new motifs that were conserved in SPDS, SPMS and ACL5. Analysis of the promoter elements revealed the incidence of conserved elements (STRE, CAAT-box, TATA-box, and MYB TF) in all promoters and highly conserved CREs in >80% of promoters (G-Box, ABRE, TGACG-motif, CGTCA-motif, as1, and MYC). The results of the quantification of PAs revealed higher levels of putrescine (Put) in the leaves and higher spermidine (Spd) in the other tissues. However, no spermine (Spm) was detected in the roots. Drought stress elevated Put level in the roots and the Spm in the leaves, shoots and roots, while decreased Put in spikes and elevated the total PAs levels in all tissues. Interestingly, PA biosynthesis genes showed tissue-specificity and some homoeologs of the same gene family showed differential gene expression during wheat development. Additionally, gene expression analysis showed that ODC is the Put biosynthesis path under drought stress in roots.

**Conclusion:**

The information gained by this research offers important insights into the transcriptional regulation of PA biosynthesis in wheat that would result in more successful and consistent plant production.

**Supplementary Information:**

The online version contains supplementary material available at 10.1186/s12864-022-08946-2.

## Background

Polyamines (PAs) are small molecules that function as secondary messengers in signalling pathways [1, 2] and are commonly distributed in eukaryotic and prokaryotic cells [3, 4]. Putrescine (Put) is a diamine which contains two amino groups, spermidine (Spd) is a triamine, and spermine (Spm) is a tetraamine which are major PAs and are primarily present in free form and play key roles in various physiological processes such as organogenesis, embryogenesis, fruit ripening, senescence, flower and cereal development [4–7]. Thermospermine (Tspm) and cadaverine (Cad) are minor PAs that are induced by limited conditions [8, 9]. PAs are also implicated in responses to different elicitors such as drought, nutrient deficiency, salinity, heat and low temperature [1, 8, 10] and at least one of these three PAs significantly increases after being subjected to stress conditions [4, 10].

**Fig. 1:**
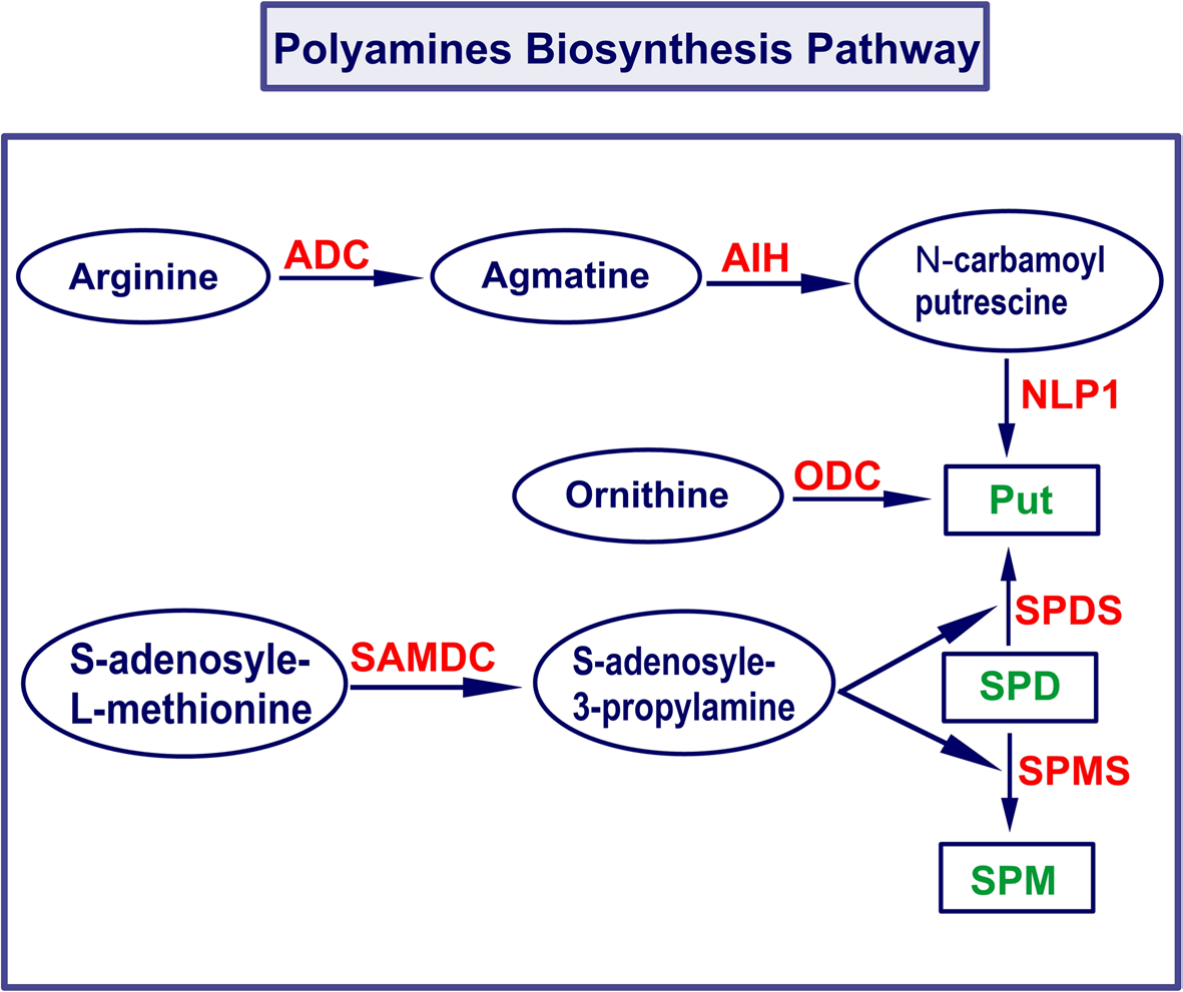
The PAs synthesis pathway. The green colour is the PA compounds. The molecules encircled in blue lines represent the precursor or substrate for PAs biosynthesis by PA synthesizing enzymes (written in red). ACL5, thermospermine synthase; ADC, arginine decarboxylase; AIH, agmatine iminohydrolase; ODC, ornithine decarboxylase; NLP1, N-carbamoylputrescine amidohydrolase/Nitrlase like protein 1; SAMDC, S-adenosylmethionine decarboxylase; SPDS, spermidine synthase; SPMS, spermine synthase

Plant PAs vary in their distribution within plant tissues and even within cell compartments. Takahashi et al. [11] discovered that leaves accumulate Put while other organs accumulate Spd. In the carrot cells, Put showed cytoplasmic accumulation whereas Spm was linked to the cell wall [12]. The accumulation or the extensive variation in PAs distribution patterns at certain stages or in definite organs recommends that regulation of PAs functions through controlling the level of PAs plus the expression of their biosynthesis genes accordingly. Alcazar et al. [13] have reported the biosynthesis path of PAs in *Arabidopsis thaliana*. The Put (diamine) is the first PA synthesized by decarboxylation of arginine (Arg) through arginine decarboxylase (ADC), additionally to agmatine iminohydrolase (AIH) and N-carbamoylputrescine amidohydrolase/Nitrlase like protein 1 (CPA/NPL1) activities in additional successive steps (Fig. 1). Put is then converted into Spd by Spd synthase (SPDS) or Spm by Spm synthase (SPMS) by adding aminopropyl groups and using SAM-decarboxylase (SAMDC) to produce decarboxylated-S-adenosylmethionine (dcSAM).

Wheat is a main cereal crop in many countries and is an essential source of nutrients for around 40% of the world’s population. Despite the importance of PAs in plant growth and stress resistance [10, 14–17], there have been no in-depth studies to dissect the polyamines biosynthesis at the genomic and transcriptional levels in wheat until now. Our previous study [10] showed that drought stress upregulated PAs and their biosynthesis genes in wheat seedlings. However, no other study investigated the transcriptional profile in wheat tissues during growth stages. In this context, the aims of the current research were to: 1. completely identify and characterize wheat genes implicated in PA biosynthesis; 2. analyse the *Cis*-elements in the promoter regions of these genes; and 3. assess the transcriptional regulation of PA biosynthesis genes in different tissues during wheat growth stages in control and drought-treated plants.

## Results

### Gene identification of PA biosynthesis genes

**Fig. 2:**
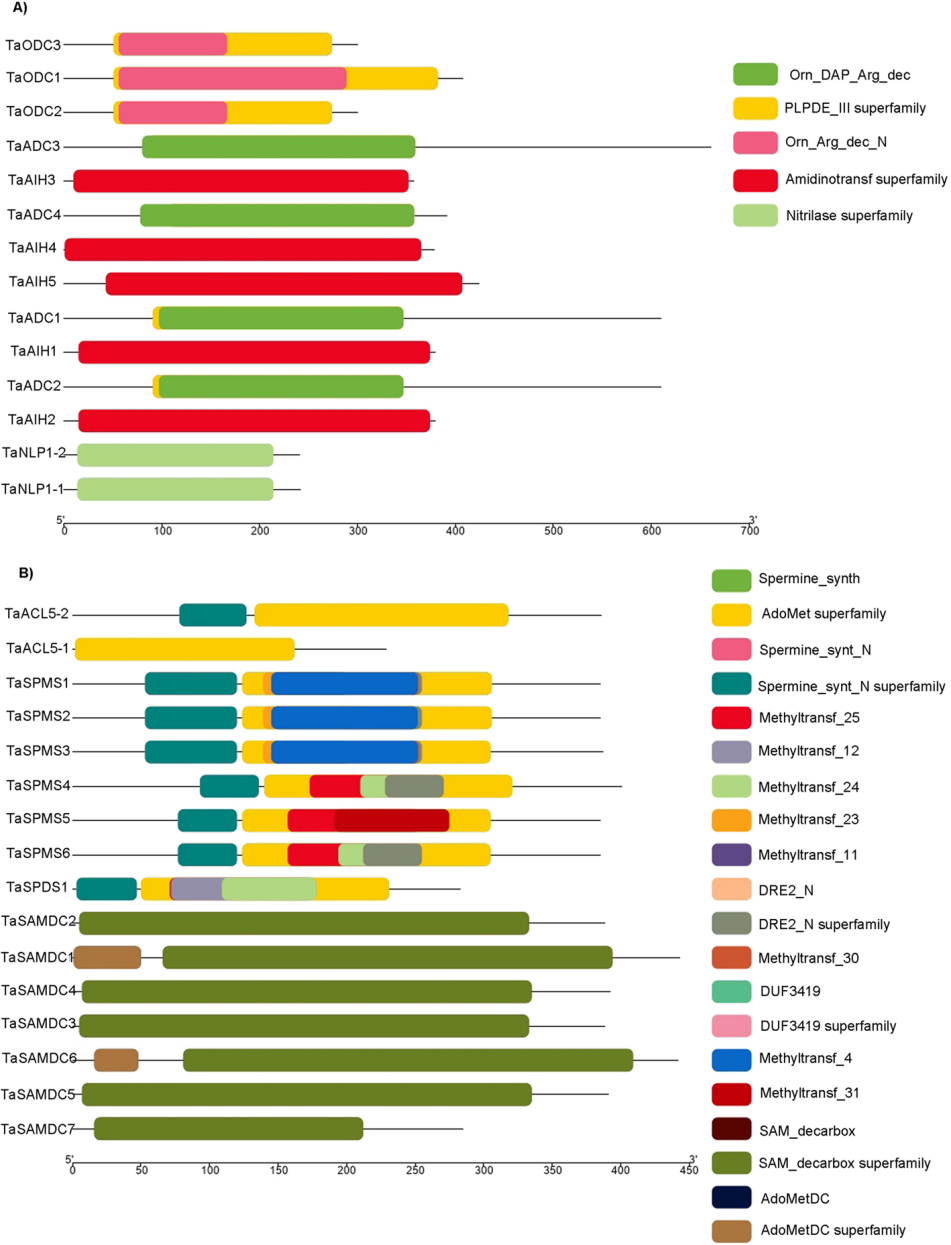
Distribution of conserved domains in Put biosynthesis proteins (ADC, ODC, AIH and NLP1); **A** and domains in SPD, SPM and TSPM biosynthesis proteins (SAMDC, SPDS, SPMS and ACL5); **B**

Genes encoding PAs biosynthesis proteins were identified using 11 *A. thaliana* protein sequences (Table 1). These genes corresponded to eight gene families (arginine decarboxylase; *ADC*, agmatine iminohydrolase; *AIIH*, nitrilase-like protein 1; *NLP1*, S-adenosylmethionine decarboxylase; *SAMDC*, spermidine synthase; *SPDS*, spermine synthase; *SPMS*, and acaulis 5; *ACL5*) in addition to ornithine decarboxylase; *ODC* “which is absent from the *A. thaliana* genome”. Based on the blast analysis, a total of 50 non-redundant wheat homologs were obtained (Supplementary Table S1). Protein domains for *A. thaliana* were identified, then compared with the identified domains in the retrieved sequences from the Blast result using CD analysis at NCBI, SMART, and Pfam (Supplementary Table S2, S3, S4). Short protein sequences and sequences with incomplete N- or C-terminus were excluded to give a total of 30 putative gene members in wheat encoding PA biosynthesis proteins containing the same corresponding domains of PA biosynthesis proteins in *A. thaliana* (Fig. 2). The *A. thaliana* genome contains two *ADC* and no *ODC*, whereas four *ADC* genes (named *TaADC1*, *TaADC2*, *TaADC3* and *TaADC4*) and three *ODC* genes (given the names *TaODC1*, *TaODC2* and *TaODC3*) were identified in wheat. Each of the *AIH*, *NLP1*, *SPMS*, and *ACL5* genes has only one locus in *Arabidopsis* but two or more homologs in wheat. Likewise, *Arabidopsis* has four *SAMDC* whereas, six members were found in wheat (Table 1).

**Table 1:** Description of the identified homologs of PAs biosynthesis genes in the wheat genome

Gene Name	AGI	Symbol	Gene ID (IWGSC v1.2)	Location	Strand	No. of AAs	Mol. Wt. KDa	pI	Instability index
Arginine decarboxylase	AT2G16500	*TaADC1*	*TraesCS1B02G018200*	1B:8772543–8775516	reverse	610	66.5	6.3	30.01
	AT4G34710	*TaADC2*	*TraesCS1D02G012300*	1D:6475182–6477014	forward	610	66.5	6.55	29.81
		*TaADC3*	*TraesCSU02G047800*	Un:37811053–37813077	reverse	601	66.1	6.8	36.68
		*TaADC4*	*TraesCS2A02G071200*	2A:31694887–31697036	forward	391	42.8	8.98	38.55
Ornithine decarboxylase	absent	*TaODC1*	*TraesCS5B02G336200*	5B:519231188–519232830	reverse	408	43.5	5.54	33.52
		*TaODC2*	*TraesCS5B02G304100*	5B:488125651–488126880	reverse	300	32.7	5.37	45.1
		*TaODC3*	*TraesCS5B02G304200*	5B:488190034–488191608	forward	300	NP	NP	42.67
Agmatine iminohydrolase	AT5g08170	*TaAIH1*	*TraesCS2D02G328900*	2D:422079023–422085254	forward	379	42.2	5.25	40.15
		*TaAIH2*	*TraesCS2A02G334600*	2A:568399774–568405206	forward	379	42.2	5.25	39.83
		*TaAIH3*	*TraesCS2B02G347800*	2B:494371121–494376445	forward	357	39.5	5.12	36.22
		*TaAIH4*	*TraesCS5A02G195100*	5A:398286820–398290961	reverse	378	42.0	5.31	40.78
		*TaAIH5*	*TraesCS5D02G198300*	5D:301125775–301129288	reverse	424	46.8	5.9	40.55
N-carbamoyl putrescine amidohydrolase	AT2G27450	*TaNLP1–1*	*TraesCS5B02G022300*	5B:20829916–20833469	forward	242	27.1	5.57	29.1
		*TaNLP1–2*	*TraesCS5A02G024500*	5A:19223045–19227613	forward	241	27.0	5.57	28.38
S-adenosylmethionine decarboxylase	AT3G02470	*TaSAMDC1*	*TraesCS6A02G219500*	6A:407085609–407086775	forward	443	48.3	5.11	44.57
	AT5G15950	*TaSAMDC2*	*TraesCS6D02G202500*	6D:285869726–285870892	forward	388	42.2	4.93	38.46
	AT3G25570	*TaSAMDC3*	*TraesCS6B02G249000*	6B:446836280–446837571	forward	388	42	4.79	38.82
	AT5G18930	*TaSAMDC4*	*TraesCS2A02G355400*	2A:598161186–598162492	reverse	392	42.9	4.97	40.27
		*TaSAMDC5*	*TraesCS2B02G372900*	2B:531466716–531467891	forward	391	42.7	4.92	39.43
		*TaSAMDC6*	*TraesCS5B02G220000*	5B:394266417–394267595	forward	442	47.8	5.23	51.12
		*TaSAMDC7*	*TraesCS5D02G228900*	5D:336223123–336224301	forward	285	NP	NP	55.12
Spermidine synthase	AT1G23820	*TaSPDS*	*TraesCS7B02G163500*	7B:225507362–225508913	forward	283	31.4	5.12	48.32
	AT1G70310								
Spermine synthase	AT5G53120	*TaSPMS1*	*TraesCS5A02G172600*	5A:365667504–365676095	reverse	385	41.9	5.56	44.15
		*TaSPMS2*	*TraesCS5B02G169900*	5B:314299114–314308267	reverse	385	41.9	5.3	45.62
		*TaSPMS3*	*TraesCS5D02G177100*	5D:276935715–276944280	reverse	387	42.3	5.74	39.33
		*TaSPMS4*	*TraesCS7B02G232700*	7B:436081229–436087600	forward	401	44.7	8.23	43.87
		*TaSPMS5*	*TraesCS7A02G350100*	7A:512759964–512765806	reverse	385	42.1	6.04	40.22
		*TaSPMS6*	*TraesCS7D02G328500*	7D:420087353–420091292	forward	385	42	6.04	38.93
ACAULIS 5	AT5G19530	*TaACL5–1*	*TraesCS7D02G094300*	7D:56880436–56886021	forward	386	43.7	6.18	35.67
		*TaACL5–2*	*TraesCS4A02G398300*	4A:673411559–673414121	forward	229	25.7	5.84	26.23

*MW* Molecular weight (Da), *pI* Isoelectric point, *NP* Not predicted as the sequences contain numerous undefined AA, so pI and Mw cannot be computed

**Fig. 3:**
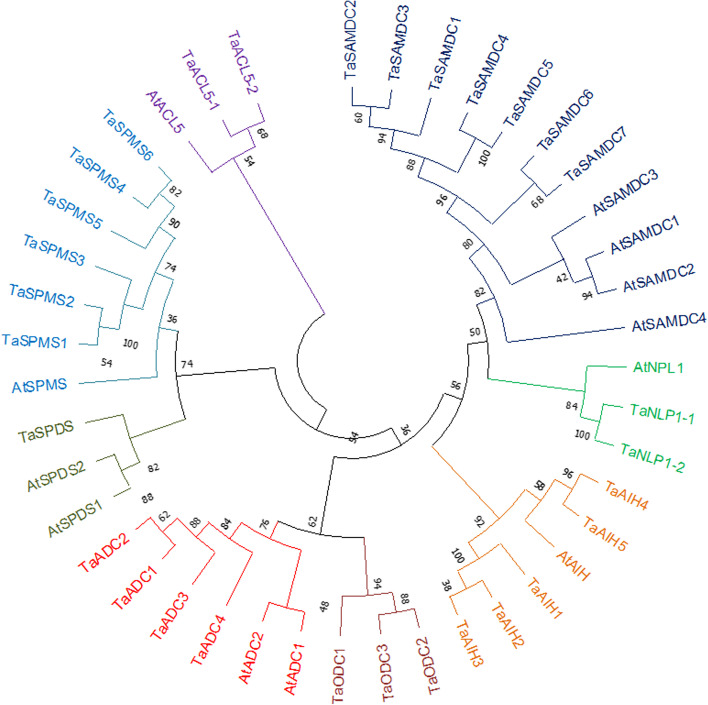
Phylogenetic analysis of proteins implicated in PA biosynthesis in wheat and *Arabidopsis*. A phylogenetic tree was created using MEGA11 software (Kumar and others 2016). At, *Arabidopsis thaliana*; Ta, *Triticum aestivum*. ACL5, thermospermine synthase; ADC, arginine decarboxylase; AIH, agmatine iminohydrolase; ODC, ornithine decarboxylase; NLP1, N-carbamoylputrescine amidohydrolase/Nitrlase like protein 1; SAMDC, S-adenosylmethionine decarboxylase; SPDS, spermidine synthase; SPMS, spermine synthase

A phylogenetic tree was constructed for the identified PA biosynthesis proteins in wheat and the corresponding orthologous genes in *A. thaliana* (Fig. 3). Phylogenetic analyses revealed that the identified protein sequences are close to those in *Arabidopsis*.

### Structural characterization of wheat PA biosynthesis genes

The physical and chemical properties predicted by the Expassy online tool for 30 wheat PA biosynthesis proteins are shown in Table 1. The TaADC proteins have a maximum length of 610 amino acids (AAs) and a minimum of 391 AAs, with isoelectric points (pI values) ranging from 6.3 to 8.9 (TaADC4). The molecular weights (Mw) range from 42.8 to 66.5 kDa. The three TaODC proteins have a maximum length of 408 AAs and the shortest of 300 AAs-residues. The pI of TaODC1 and TaODC2 were 5.4 and 5.3, respectively, and the Mw were 43.5 kDa (TaODC1) and 32.7 kDa (TaODC2). The protein sequence of TaODC3 contained several undefined AAs so, its Mw and pI could not be computed. The analysis of AIH in wheat revealed 5 homoeologs with AAs length ranging from 357 to 424, and PI values ranged from 5.1 to 5.9 and Mw of 39.5 kDa to 46.8 kDa. The TaNLP1 proteins (TaNLP1–1 and TaNLP1–2) had 242 and 241 AAs, respectively, and 5.6 pI value, and the Mw was about 27 kDa. The SPD, SPM, and TSPM biosynthesis proteins SAMDC, SPDS, SPMS, and ACL5 had proteins with a maximum length of 443 AAs and the shortest of 229 AAs, and pI values ranged from 4.8 to 8.2 and Mw. ranged from 25.7 kDa to 48.3 kDa. Among the 30 PA biosynthesis proteins in wheat, 14 proteins showed an instability index greater than 40 (Table 1), which is considered an unstable protein [18].

**Fig. 4:**
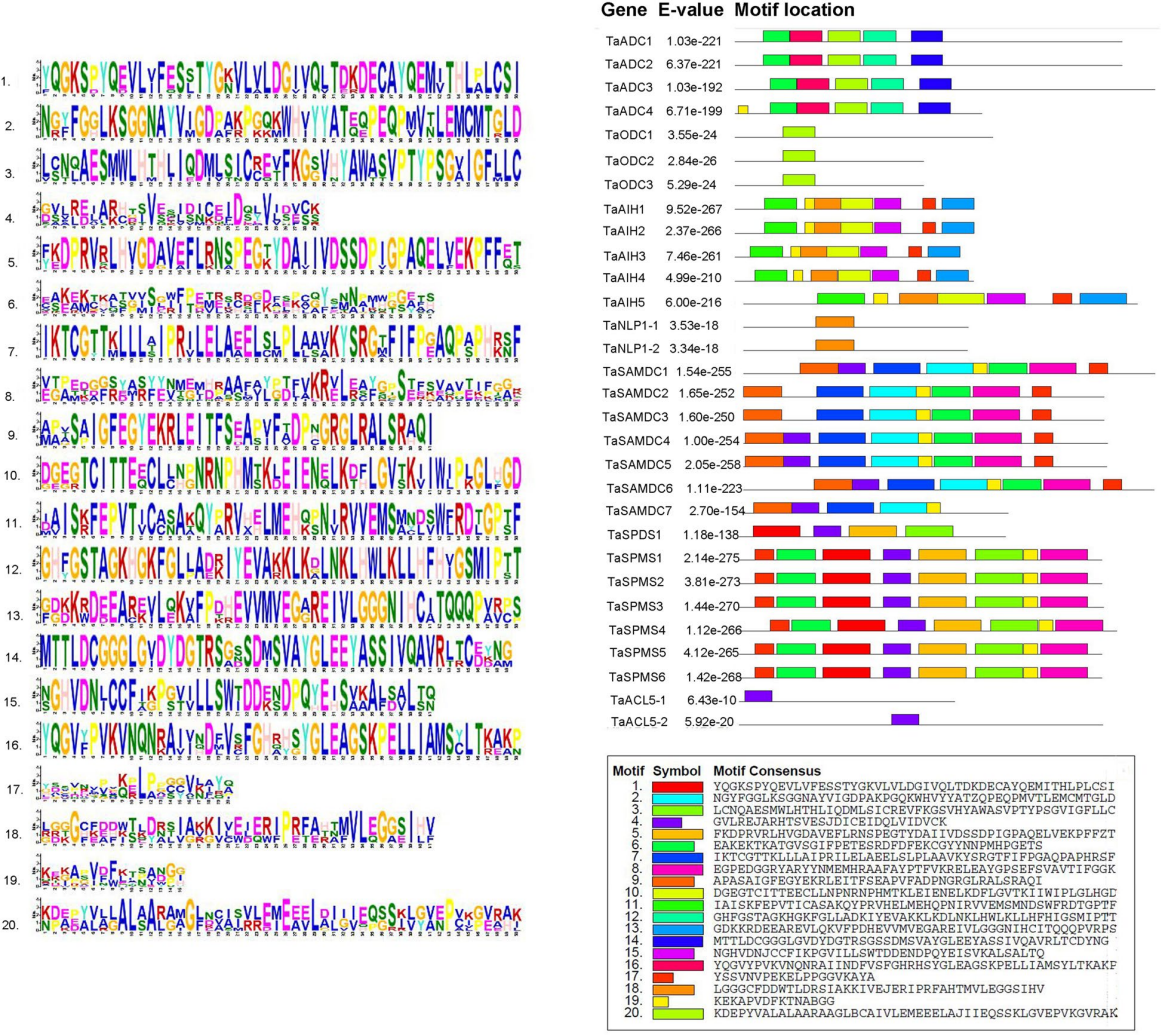
Distribution of conserved motifs. Conserved motifs (left) and their distributions (right) of TaPA biosynthesis proteins predicted by the MEME (Multiple Expectation Maximization for Motif Elicitation). The motifs are represented by different coloured boxes with a corresponding number. The descriptions of the motifs are listed in Table 2

The MEME software was used for protein sequence analysis to understand the diversity and similarity of protein motifs within each gene family. Twenty conserved motifs were discovered (Table 2). The results indicated that there were conservative motifs specific to each group. The composition of structural motifs was diverse among different groups, but similar within the same group (Fig. 4). All TaADC proteins had five motifs involving motif 11, 12, 14, 16 and 20, which correspond to Pyridoxal-dependent decarboxylase, pyridoxal binding domain (Orn_Arg_deC_N family) and *Porphyromonas*-type peptidyl-arginine deiminase (PAD_porph). The three TaODc had one conserved motif (motif20, Orn_Arg_deC_N family). The five TaAIH homologous proteins had seven conserved motifs (motif 1, 10, 11, 13, 15, 18 and 19), including five *Porphyromonas*-type peptidyl-arginine deiminase motifs and one novel motif (motif 19). The descriptions of all identified motifs are shown in Table 2. Both TaNPL1–1 and TaNLP1–2 proteins had only the motif 18 which belongs to *Porphyromonas*-type peptidyl-arginine deiminase. Among the seven TaSAMDC proteins, five proteins had eight conserved motifs (motif 2, 6, 7, 8, 9, 17 and 19). The SAMDC2 and SAMDC3 missed the motif 4. And the Adenosylmethionine decarboxylase motifs (SAM decarboxylase family) were involved in all sequences. TaSPDS protein contained four motifs for “Spermidine synthase domain” (motif 1, 3, 4 and 5). All TaSPMS sequences had eight motifs, including the SPDS motifs, in addition to motif 6, 16, 17 and 19. TSPMS had only a single motif (motif4) for spermine synthesis. Interestingly, two motifs (motif17; YSSVNVPEKELPPGGVKAYA and motif19; KEKAPVDFKTNABGG) were not found in the Pfam or any motif identifier database. These two motifs were conserved in TaAIH, TaSAMDC, and TaSPMS proteins and they may be novel motifs for these proteins (Table 2).

**Table 2:** Description of the conserved motifs identified by MEME in the PA biosynthesis proteins

Motif No.	Motif sequence	Width	Description	Family
1	YQGKSPYQEVLVFESSTYGKVLVLDGIVQLTDKDECAYQEMITHLPLCSI	50	Spermidine synthase tetramerisation domain	Spermine_synt_N
2	NGYFGGLKSGGNAYVIGDPAKPGQKWHVYYATZQPEQPMVTLEMCMTGLD	50	Adenosylmethionine decarboxylase	SAM_decarbox
3	LCNQAESMWLHTHLIQDMLSICREVFKGSVHYAWASVPTYPSGVIGFLLC	50	Spermine/spermidine synthase domain	Spermine_synth
4	GVLREJARHTSVESJDICEIDQLVIDVCK	29	Spermine/spermidine synthase domain	Spermine_synth
5	FKDPRVRLHVGDAVEFLRNSPEGTYDAIIVDSSDPIGPAQELVEKPFFZT	50	Spermine/spermidine synthase domain	Spermine_synth
6	EAKEKTKATGVSGIFPETESRDFDFEKCGYYNNPMHPGETS	41	Adenosylmethionine decarboxylase	SAM_decarbox
7	IKTCGTTKLLLAIPRILELAEELSLPLAAVKYSRGTFIFPGAQPAPHRSF	50	Adenosylmethionine decarboxylase	SAM_decarbox
8	EGPEDGGRYARYYNMEMHRAAFAYPTFVKRELEAYGPSEFSVAVTIFGGK	50	Spermine/spermidine synthase domain	Spermine_synth
9	APASAIGFEGYEKRLEITFSEAPVFADPNGRGLRALSRAQI	41	Adenosylmethionine decarboxylase	SAM_decarbox
10	DGEGTCITTEECLLNPNRNPHMTKLEIENELKDFLGVTKIIWIPLGLHGD	50	Porphyromonas-type peptidyl-arginine deiminase	PAD_porph
11	IAISKFEPVTICASAKQYPRVHELMEHQPNIRVVEMSMNDSWFRDTGPTF	50	Porphyromonas-type peptidyl-arginine deiminase	PAD_porph
12	GHFGSTAGKHGKFGLLADKIYEVAKKLKDLNKLHWLKLLHFHIGSMIPTT	50	Pyridoxal-dependent decarboxylase, pyridoxal binding domain	Orn_Arg_deC_N
13	GDKKRDEEAREVLQKVFPDHEVVMVEGAREIVLGGGNIHCITQQQPVRPS	50	Porphyromonas-type peptidyl-arginine deiminase	PAD_porph
14	MTTLDCGGGLGVDYDGTRSGSSDMSVAYGLEEYASSIVQAVRLTCDYNG	49	Pyridoxal-dependent decarboxylase, pyridoxal binding domain	Orn_Arg_deC_N
15	NGHVDNJCCFIKPGVILLSWTDDENDPQYEISVKALSALTQ	41	Porphyromonas-type peptidyl-arginine deiminase	PAD_porph
16	YQGVYPVKVNQNRAIINDFVSFGHRHSYGLEAGSKPELLIAMSYLTKAKP	50	Pyridoxal-dependent decarboxylase, pyridoxal binding domain	Orn_Arg_deC_N
17	YSSVNVPEKELPPGGVKAYA	20	Novel motif	
18	LGGGCFDDWTLDRSIAKKIVEJERIPRFAHTMVLEGGSIHV	41	Porphyromonas-type peptidyl-arginine deiminase	PAD_porph
19	KEKAPVDFKTNABGG	15	Novel motif	
20	KDEPYVALALAARAAGLBCAIVLEMEEELAJIIEQSSKLGVEPVKGVRAK	50	Pyridoxal-dependent decarboxylase, pyridoxal binding domain	Orn_Arg_deC_N

**Fig. 5:**
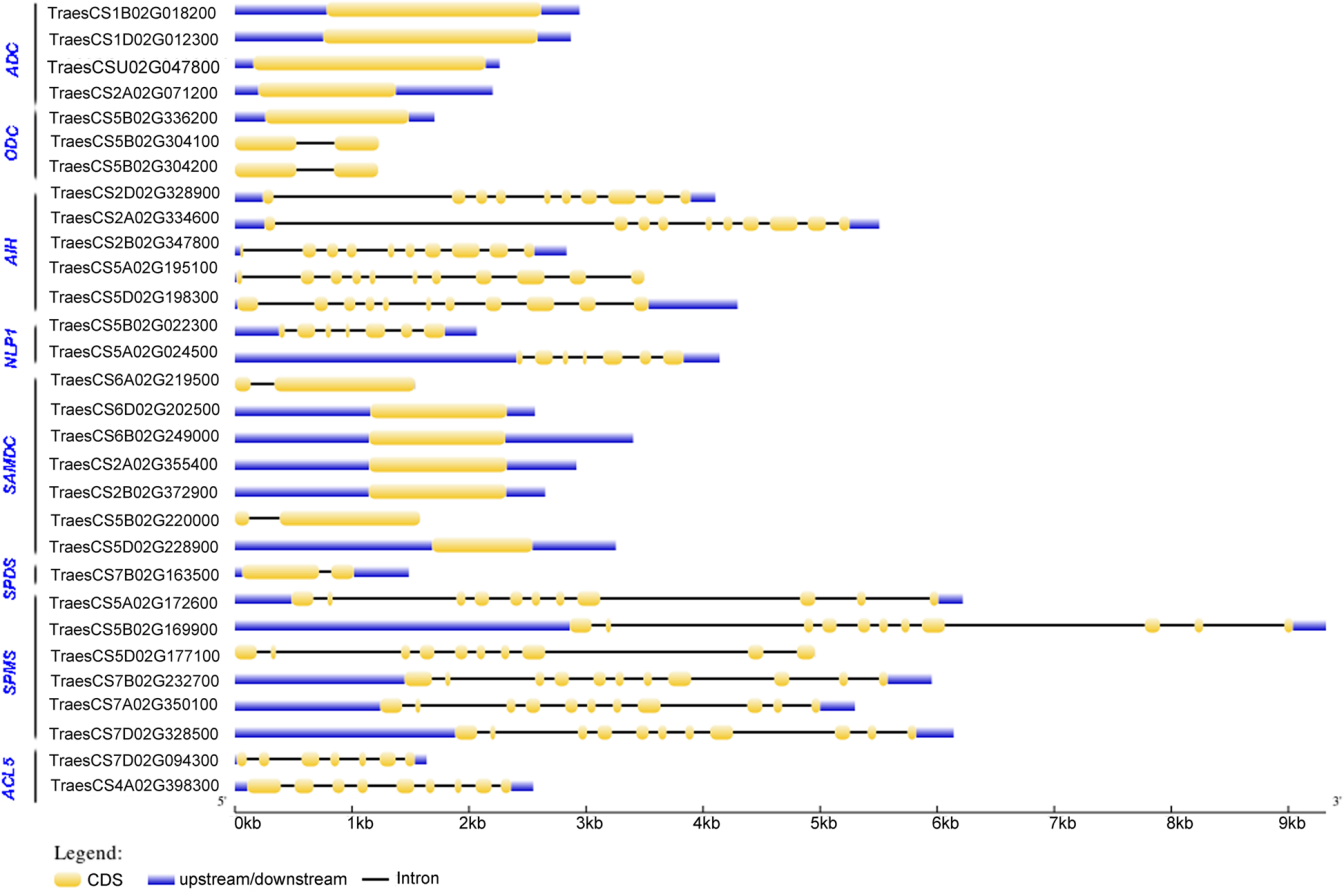
Exon–intron structure of wheat genes. The yellow blocks indicate the CDS (coding sequences), the blue blocks represent the region up- or down-stream of the genes, and the black lines represent the introns. The length of DNA sequences was indicated by a scale bar

Analysis of exon-intron structure showed that PA biosynthesis genes either contain no introns or multi-introns (up to 10 introns) (Fig. 5). The Put biosynthesis genes *ADC*, *ODC*, *AIH* and *NLP1* had different gene structures. None of the *TaADC* genes had any introns in their genomic sequences. One of the three *TaODC* genes had no introns (*TaODC1*), while the other two genes had a single intron in their genomic sequences. All the *TaAIH* and *TaNLP1* genes had multiple introns. Focusing on the gene structure of SPD, SPM, and TSPM biosynthesis genes, *SAMDC*, *SPDS*, *SPMS*, and *ACL5* have been analyzed. Most members of *TaSAMDC* had no introns in their genomic sequences, whereas *TaSAMDC1* and *TaSAMDC6* had only one intron. The SPDS encoding gene had a single intron while all the six *SPMS* genes had multi-introns. *TaACL5* had 6–8 introns (Fig. 5).

### Protein subcellular localization

The subcellular localization of wheat PAs biosynthesis proteins was predicted using several localization predictors (Supplementary Table S5). The predicted locations of the PA biosynthesis proteins were also verified by the prediction of targeting signals for chloroplasts, mitochondria, and transmembrane proteins. All members of TaADC, TaNLP1, TaSPDS and TaACL5 were predicted to localize in the cytoplasm. ODC localized to the chloroplast and verified by the existence of a plastid targeting signal. Four wheat AIH proteins were anticipated to be cytoplasmic while the fifth was expected to localize in the mitochondria and was verified by the existence of a mitochondrial targeting signal. The TaSAMDC sequences were predicted to be located in the nucleus and the chloroplast, while the TaSPMS members were predicted to be located in the cytoplasm and one member to be located in the chloroplast. Interestingly, the predicted localization for wheat proteins showed more than 90% homology predictions with *Arabidopsis* proteins. These detailed predictions are provided in Table 3.

**Table 3:** The significant prediction of subcellular localization for Arabidopsis and wheat PA biosynthesis proteins

Gene Name	AGI	Localization	Wheat gene	Localization
Arginine decarboxylase	*AtADC1*	plastid	*TaADC1*	Cytoplasm
	*AtADC2*	plastid	*TaADC2*	Cytoplasm
			*TaADC3*	Cytoplasm
			*TaADC4*	Chloroplast
Ornithine decarboxylase	Absent	–	*TaODC1*	Chloroplast
			*TaODC2*	Chloroplast
			*TaODC3*	Chloroplast
Agmatine iminohydrolase	*AtAIH*	cytosol	*TaAIH1*	Cytoplasm
			*TaAIH2*	Cytoplasm
			*TaAIH3*	Cytoplasm
			*TaAIH4*	Cytoplasm
			*TaAIH5*	Mitochondria
N-carbamoyl putrescine amidohydrolase	*AtNLP1*	cytosol	*TaNLP1–1*	Cytoplasm
			*TaNLP1–2*	Cytoplasm
S-adenosylmethionine decarboxylase	*AtSAMDC1*	plasma membrane	*TaSAMDC1*	Nucleus
	*AtSAMDC2*	cytosol	*TaSAMDC2*	Nucleus
	*AtSAMDC3*	cytosol	*TaSAMDC3*	Nucleus
	*AtSAMDC4*	cytosol	*TaSAMDC4*	Chloroplast. Nucleus
			*TaSAMDC5*	Chloroplast. Nucleus
			*TaSAMDC6*	Nucleus
			*TaSAMDC7*	Nucleus
Spermidine synthase	*ATSPDS1,2*	cytosol	*TaSPDS*	Cytoplasm
Spermine synthase	*AtSPMS*	cytosol	*TaSPMS1*	Cytoplasm
			*TaSPMS2*	Cytoplasm
			*TaSPMS3*	Cytoplasm
			*TaSPMS4*	Chloroplast
			*TaSPMS5*	Cytoplasm
			*TaSPMS6*	Cytoplasm
ACAULIS 5	*AtACL5*	cytosol	*TaACL5–1*	Cytoplasm
			*TaACL5–2*	Cytoplasm

### Cis-elements in the promoters of wheat PA biosynthesis genes

**Fig. 6:**
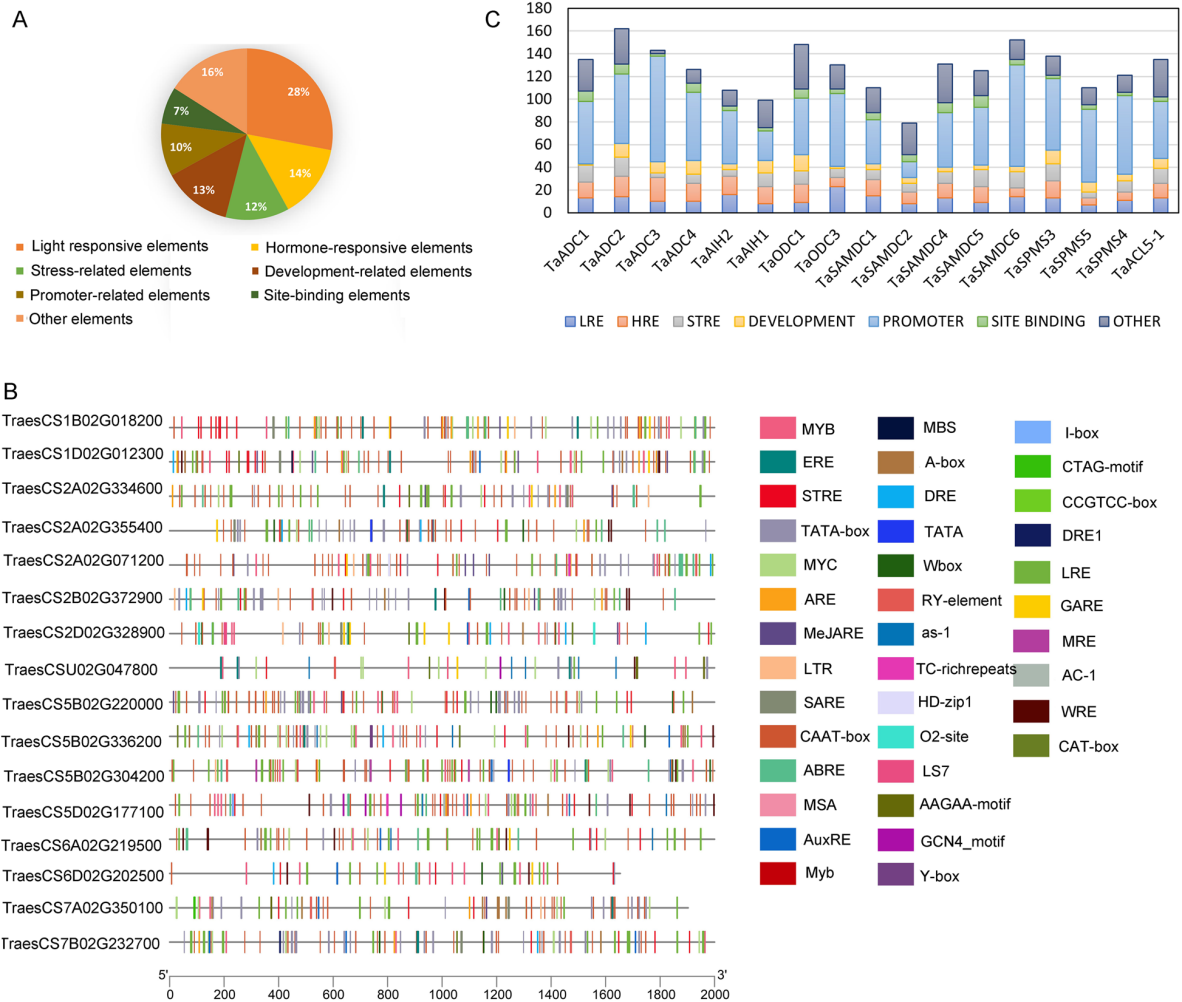
*Cis*-regulatory elements in the promoter region of PA biosynthesis genes in wheat. **A** Pie distribution of the sum of 100 identified CREs in wheat PA biosynthesis promoters classified into seven categories. **B** Position of the identified CREs in wheat 2000 bp promoter. **C** Distribution of each CREs category in each wheat promoter, number of elements of each CREs category is represented by a different colour in the histogram

**Fig. 7:**
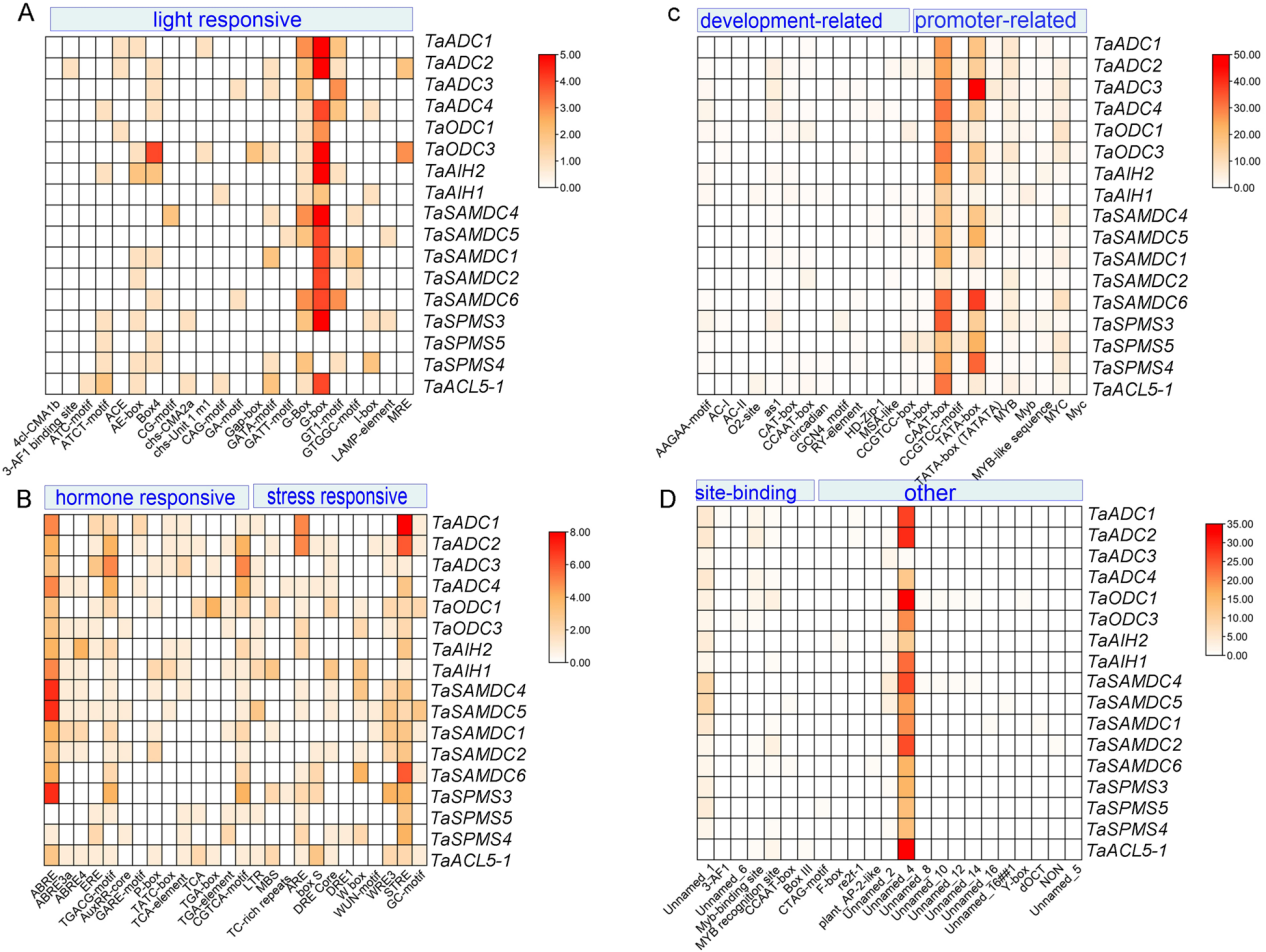
Heatmap of the CREs number in each identified wheat PA biosynthesis promoters. **A** Frequency of the light responsive CREs in wheat promoters. **B** Frequencies of hormone and stress responsive CREs. **C** The frequency of CREs related to development- and site-binding. **D** Frequencies of site-binding and other elements in wheat promoters. The numbers of CREs are represented by different colours, and the white colour indicates no CRE

*Cis*-acting elements (CREs) control gene regulation through binding with other regulatory proteins. Thus, analysis of promoter elements in PA biosynthesis genes in wheat would provide invaluable information about their regulation. The promoter sequences for all the genes were analysed by the PlantCARE database for *Cis*-element identification. In total, 100 different *Cis*-elements were identified (Supplementary Table S6). The identified CREs were categorised into seven groups; promoter-related, site-binding related (light-responsive, hormone-responsive, environmental-responsive, developmental, and other elements with unknown function (Fig. 6A). The position of the CREs in the 2000 bp promoter sequences before the ATG position of each gene was presented in (Fig. 6B). All CREs categories were represented in all wheat promoters but with different frequencies (Fig. 6C). The number and percentage of the putative CREs of PA biosynthesis gene promoters in wheat are summarized in Supplementary Table (S7). Five CREs were noticed in all promoters (Fig. 7). These are one stress-responsive element (STRE), three promoter-related elements; CAAT-box, TATA-box, MYB transcription factor, and one unknown function (Unnamed-4). Furthermore, CREs involved in light responses (G-Box and G-box), CRE involved in the response to abscisic acid (ABRE), CREs involved in the methyl jasmonate (MeJA)-responsiveness (TGACG-motif and CGTCA-motif), CRE involved in tissue-specific expression (as1), a transcription factor (MYC), and 60 K protein binding site (Unnamed-1) were the most common (>80%) in all promoters.

### Expression profiles of PA biosynthesis genes in different tissues and stages

**Fig. 8:**
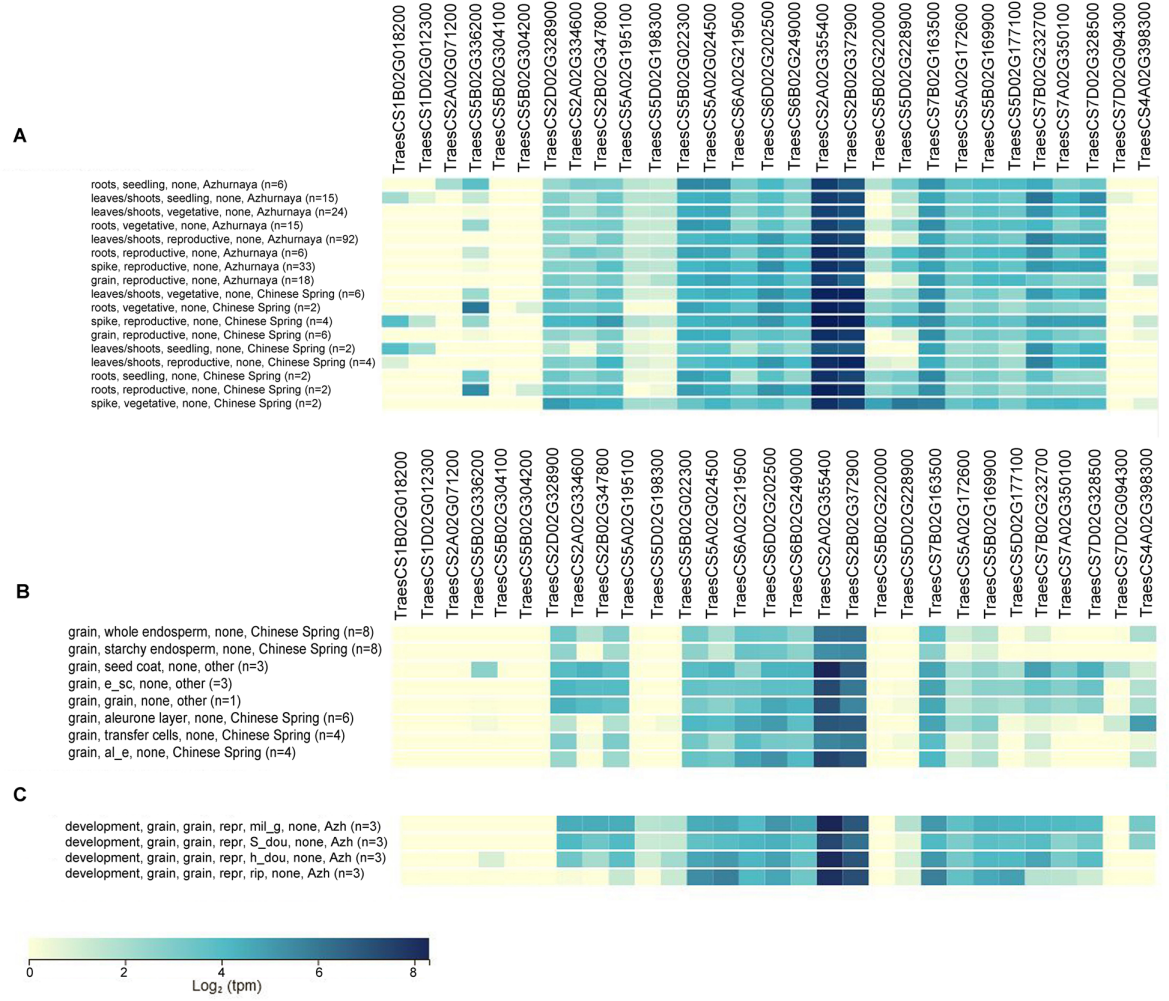
Transcriptional profiles of wheat PAs biosynthesis in various tissues by global transcriptome analysis. The relative expression data is TPM (Transcripts Per Million). **A** a heatmap of gene expression in different tissues at various stages of development. **B** a heatmap for gene expression in dissected wheat grain tissues. **C** a heatmap of gene expression in wheat grain at various stages of development. Blue-coloured blocks indicate lower and higher transcription levels corresponding to the colour-key

To examine the importance of PAs biosynthesis regulation in wheat development, their gene expression was analysed using publicly available data (Fig. 8).

To explore the transcriptional responses at different stages, data from two time-course experiments (the developmental time-course of Chinese spring and Azhurnaya) were chosen for analysis and presented as a heatmap (Fig. 8A). Notably, some genes were highly-expressed at all stages (seedling, vegetative and reproductive stage) while others showed expression at certain growth stage and three genes were not expressed at all (*TaODC2, TaODC3*; *TraesCS5B02G304100*, *TraesCS5B02G304200* and *TaACL5–1*; *TraesCS7D02G094300*). The highest expression was for *TaSAMDC4*, *TaSAMDC5* and *TaSPDS* (*TraesCS2A02G355400, TraesCS2B02G372900* and *TraesCS7B02G163500* respectively) at all stages. Interestingly, *TaAIH4,5* (*TraesCS5A02G195100* and *TraesCS5D02G198300*) showed very low expression at all stages and *TaACL5–2* (*TraesCS4A02G398300*) was only expressed in tissues at the reproductive stage, whereas *TaADC1,2* showed expression only in leaves and spike samples and *TaADC4* in roots only, but *TaODC1* (*TraesCS5B02G336200*) was not expressed in grain samples. Expression profiles by high-throughput sequencing of spike tissue at different times were examined. The results are found in a supplementary Table (S8). All homoeologs of *TaADC*, *TaODC* and *TaACL5* showed weak or no expression at the spike stages (vegetative, elongation, glume and floret stages) except that *TaADC2* expressed at the vegetative stage. The transcripts of *TaAIH1–3*, *TaSPDS*, *TaSPMS1–3* and *TaSAMDC3*–*4* were higher at glum and floret stages than at vegetative and elongation stages. Interestingly *TaSAMDC5* and *TaSAMDC6* showed the highest transcript levels in all stages.

For a better understanding of the transcriptional changes in wheat grains, two tissue-specific developmental time-course experiments were chosen for analysis and presented as a heatmap (Fig. 8B). The first experiment showed the transcriptional profile in dissected grain tissues (whole endosperm, starchy endosperm, seed coat, scutellum, and aleurone layer) (Fig. 8B). *TaODC1* showed expression only in the seed coat and some genes (*TaADC1, 2, 3*; *TaODC2, 3*; *TaAIH4, 5;* and *TaSAMD6, 7*) were not expressed in any tissue at all. *TaAIH2* wasn’t expressed in the starchy endosperm and aleurone layer. *TaSPMS3*, *TaSPMS5* and *TaSPMS6* were expressed only in the scutellum and seed coat, whereas the other genes (*TaAIH1*, 3, *TaNLP*, *TaSAMDC1–5*, *TaSPDS* and *TaSPMS2*) were expressed in the whole dissected grain tissues. The second experiment showed a heatmap for gene expression levels in grains at different phases of the grain filling period (milky stage, soft dough, hard dough, and ripening) (Fig. 8C). As displayed in the figure, eight genes were not expressed at any stage of grain filling (*TaADC1*, 2, 3; *TaODC1, 2, 3; TaSAMD6* and *TaACL5–1*). *TaACL5–2* expressed at the milky and soft dough stages only, whereas all *TaAIH* members except *TaAIH5* showed expression at all stages except the ripening stage.

### PA content and gene expression profiles of their biosynthesis genes in drought-stressed wheat

**Fig. 9:**
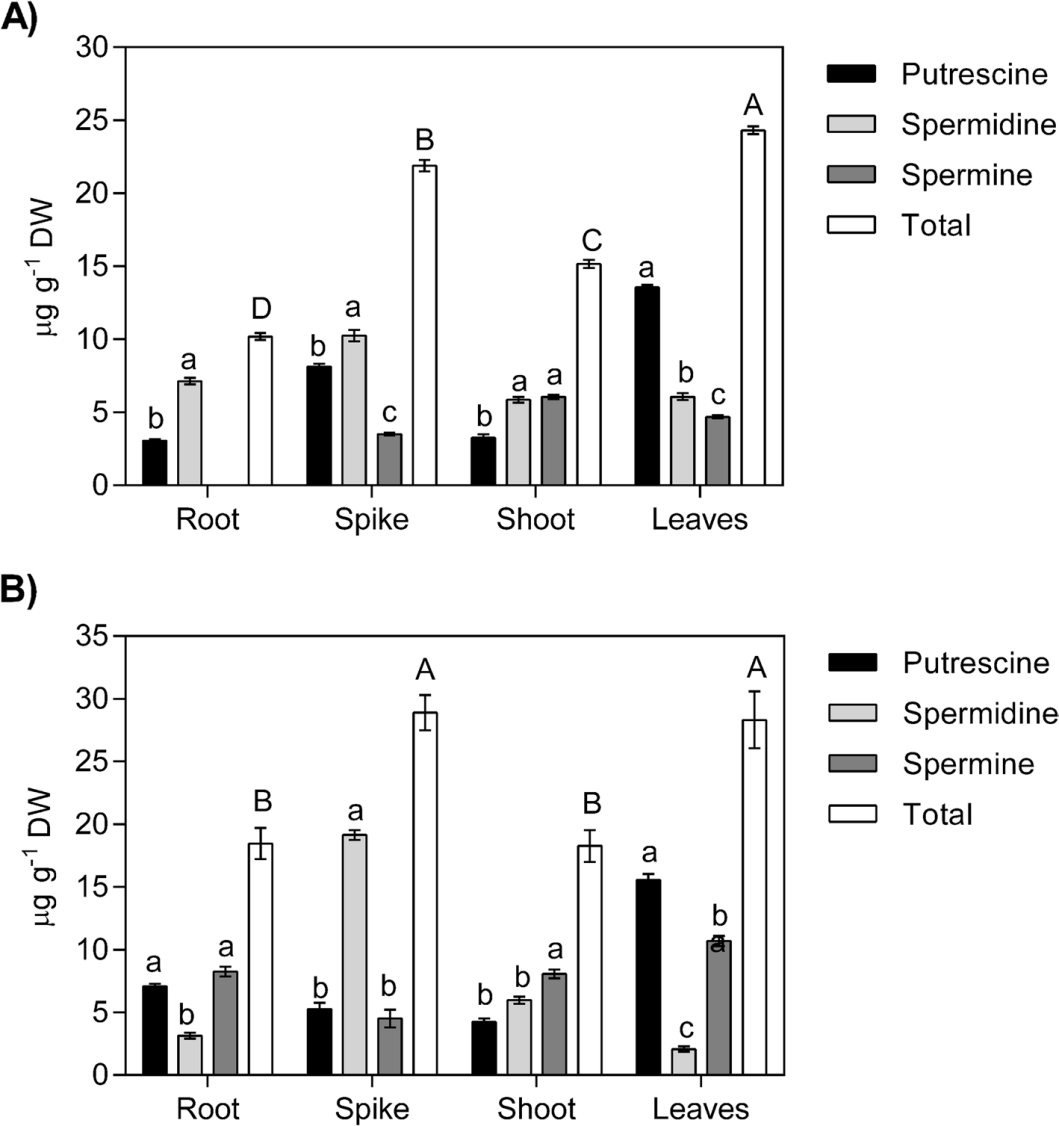
Quantification of polyamines (PAs: Putrescine, Put; Spermidine, Spd; Spermine, Spm) in various wheat tissues (spike, shoot, leaves, and roots) of an adult plant (45DAE). **A** PAs concentration (μg g^−1^) under normal conditions. **B** PAs concentration (μg g^−1^) under drought conditions. The data represents the mean of three biological replicates. Different letters on bars indicate significance at *P* ≤ 0.05 (small letters for comparison of individual PAs within the same tissue and capital letters for comparison of total PAs in different tissues)

To investigate PAs content in wheat tissues under irrigated and drought conditions, samples from leaves, shoot axis, spike and roots were analysed. Quantification of PAs revealed that Put, Spd, and Spm were differentially distributed in the whole tissues and that Spm was not detected in the roots under irrigated conditions (Fig. 9A). The results revealed that Spd was the most abundant PA in the spike, shoot and root tissues, and Put was higher in leaves than in the other tissues, whereas the concentration of total PAs was in the following order: leaves>spike>shoot>root. Interestingly, under drought conditions, Spd was higher in spike while Put was higher in leaves, whereas Spm was elevated in shoot and root (Fig. 9B). Furthermore, Put levels in the spike decreased while they increased in the roots.

**Fig. 10:**
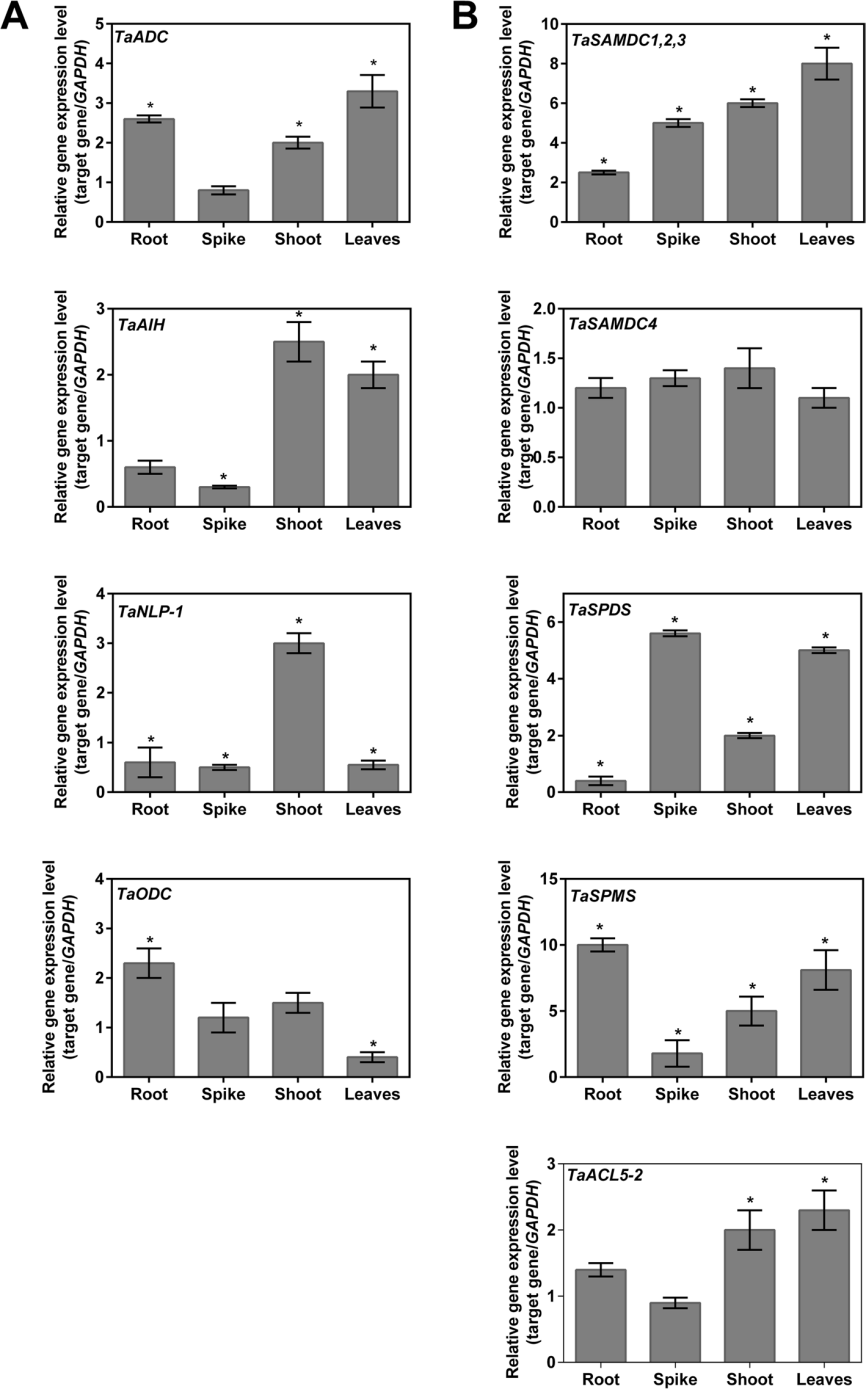
Quantitative gene expression of PAs biosynthesis under drought conditions in wheat plants (45 DAE). The panel (**A**) shows the transcriptional regulation of Put biosynthesis and the panel (**B**) shows the transcriptional regulation of Spd and Spm biosynthesis. For quantitative analysis, total RNAs from spikes, shoot axes, leaves, and leaves were used. The expression level in each sample was represented as fold change expression relative to its control expression values. Three technical and three biological replicates were used. The values are the mean ± standard error. Significance at *P* ≤ 0.05 was indicated by (*)

To validate the results of the *In-silico* gene expression analysis, the transcriptional changes of PA biosynthesis genes were evaluated in four tissues (leaves, shoot axis, spikes, and roots) in adult wheat plants (45 DAS) using qPCR under normal and drought conditions. Specific primer pairs were designed as specifically as possible, but in some cases because of the high similarity between some homoeologs, universal primer pairs were designed to quantify the total expression levels of the homoeologous genes (Supplementary Table S9). The gene expression levels of control samples were set to one, and the relative gene expression values of drought-treated samples are shown in Fig. 10. Markedly, all the investigated genes showed expression in all the examined tissues. Furthermore, genes involved in the arginine pathway for Put biosynthesis (*ADC*, *AIH*, and *NPL1*) were upregulated by drought stress in the shoots, while only the *ADC* was up-regulated in the roots and both *ADC* and *AIH* were up-regulated in the leaves (Fig. 10A). However, both *AIH*, and *NPL1* were down-regulated in the spikes by drought conditions. On contrast, stress conditions increased *ODC* expression in roots to about 2.5 folds and significantly decreased the expression level in the leaves. The total expression of *SAMDC1*, *SAMDC2* and *SAMDC3* was highly upregulated in all tissues under drought. However, *SAMDC4* was not significantly changed by stress treatment (Fig. 10B). Notably, the *SPDS* was up-regulated by drought treatment in spikes, shoots and leaves while down-regulated in roots. The *SPMS* was up-regulated by drought treatment in all tissues except the spikes. The *ACL5–2* showed significant upregulation in shoots and leaves only.

## Discussion

### Structural characterization of PA biosynthesis genes in wheat

Polyamines are ubiquitous and have been revealed to be essential for various plant developmental processes. In addition, they are commonly involved in responding to environmental stresses [10, 19, 20]. Considerable interest has been given to the diverse functions of PA in responses to stress, so the bulk of the published studies focused on using PAs as exogenous stimulants and revealed improved tolerance to various stresses [8, 10, 21]. However, the allocation of PAs in different wheat tissues and growth phases at the transcriptional level remains largely unknown under normal or stress conditions, particularly in wheat. Therefore, the goal of this investigation was to identify these genes in wheat and to comprehensively analyse their structure and transcriptional changes in various tissues under regular and drought conditions.

The biosynthesis pathways for PAs are conserved among organisms [22]. The first diamine Put is synthesized by both the ADC and ODC pathways. Then, tri- and tetra-amines (Spd and Spm) are formed by SPDS and SPMS with the assistance of SAMDC and Tspm is formed by ACL5 [23]. In this study, thirty genes implicated in PAs biosynthesis were identified and characterized with a focus on their gene structures and phylogenetic relationships in the wheat genome by a genome-wide approach. Although genes encoding PA biosynthesis in *A. thaliana* have been completely identified, the identification of these genes in other species has been partially identified. In tomato, citrus, and rice, only *ADC*, *ODC*, *SPDS*, *SPMS*, and *ACL5* were identified [24, 25], and in maize *SPMS* [26]. The current identification of PA biosynthesis genes in wheat revealed that they are distributed on 14 wheat chromosomes. Some gene families such as *TaADC* and *TaSAMDC* showed an uneven distribution across the sub-genomes A, B, and D. *TabZIP* and *TaGST* family members most likely showed unequal distribution as well [27, 28].

The occurrence of different routes for Put biosynthesis through both *ADC* and *ODC* was formerly described in different species, particularly under stressors [10, 24, 29]. Although *ODC* is absent from some species like *A. thaliana* and *Spirodela polyrhiza* [30, 31], it was identified in *Oryza sativa*, *Citrus sp.*, and *Glycine max* by only one locus [32], two in apple [33] and three in tomato [24]. Here, three *ODC* paralogs were identified in wheat on chromosome 5B whereas four *ADC* candidate genes were found on chromosomes 1B, 3B, 1D and 2A. Two orthologs of *ADC* were identified in apple and tomato [24, 33]. and three in rice [25]. The presence of both Put biosynthesis pathways in wheat strengthens the occurrence of functional regulation mechanisms.

The other involved Put biosynthesis genes through the ADC pathway; *AIH* and *CPA*/*NLP1* have not been identified in too many species. In the current study, five *TaAIH* and two *TaNPL1* were revealed by analysis, while a single *AIH* and *NPL1* were found in *S. polyrhiza* [30] and *Citrus* as in *A. thaliana* [31]. Rice showed to have one *AIH* and four *NLP1* [25].

Because the conserved domains of the tri- and tetra PAs biosynthesis proteins (SPDS and SPMS) are similar, phylogeny and motif analyses were used to identify their paralogs. Earlier, *Arabidopsis SPMS* was defined as *AtSPDS3* which revealed that *SPDS* and *SPMS* are not distinct from each other [3]. Here, one *TaSPDS*, six *TaSPMS* and two *TaACL5* were identified. This varied number of PA biosynthesis genes within species, 12 in *Arabidopsis* and 30 in wheat was expected because of the polyploidy nature of wheat and also implies the occurrence of duplication incidents during the wheat genome evolution.

### Subcellular localization of wheat proteins

Subcellular localization is crucial for understanding the functional involvement of genes. Lack of knowledge of the exact cellular compartmentation of these proteins has been a main barrier to understanding of the biological functions of PAs in plants. In this study, most wheat proteins possessed signal sequences and were predicted to be located in the plastid, mitochondria and cytoplasm (Table 3). The prediction assessment revealed that within the wheat PAs biosynthesis proteins, 17 proteins were localized to the cytosol, 4 to the chloroplast, 4 to the nucleus, one to the mitochondria, and two to the nucleus and chloroplast. ADC localizes the chloroplast in *Arabidopsis* but in wheat, it showed multi-localization in the nucleus, chloroplasts, mitochondria, peroxisomes and cytoplasm, which is in accordance with the prediction of *Malus hupehensis* [33]. ODC in wheat localized the chloroplast and cytoplasm however it is only plastidic in apple [33]. SAMDC in *Arabidopsis* has two different sub-cellular localizations (cytoplasm and plasma membrane). Likely, TaSAMDC localized different compartments (cell membrane, chloroplast, and nucleus) and this is in accord with the prediction of SAMDC in *M. hupehensis* [33]. SPMS localizes in the cytosol in *Arabidopsis* and corn [26] and the current analyses revealed that it has a similar localization in wheat. Likely, SPDS was cytosolic in wheat as in *M. hupehensis* [33]. Subcellular localization of *Arabidopsis* proteins has been predicted using SUBA, the subcellular localization database for *Arabidopsis* proteins; (www.suba.live) [34] to retrieve the most likely subcellular localization based on SUBAcon (an algorithm that integrates multiple subcellular prediction programmes and experimental data where this exists. Although that most prediction of the subcellular localization of PA biosynthesis proteins in wheat are agree with protein localization in *Arabidopsis* or other species, further experiments are needed in wheat to confirm the localization of each protein and help in understanding the functional involvement of the PA biosynthesis genes.

### Promoters sequence analysis in wheat

Scanning the promoter sequences for the existence of CREs using PlantCARE was essential to understand PA biosynthesis gene regulation. About 2000 bp-promoter regions were analyzed. The common and conserved CREs in 100% of the promoters are one stress-responsive element; STRE, three promoter-related elements; CAAT-box, TATA-box, and MYB transcription factor, and one element with unknown function; Unnamed-4. STRE is a well-recognized stress-responsive element (AAGGGG). It is responsive to different environmental elicitors and is a binding site for transcriptional activator, Msn2p/Msn4p, identified in yeast [35]. Nevertheless, to our knowledge, the trans-factors that recognize STRE in plants have not been reported. STRE is triggered by various stresses and regulates gene expression, as elucidated by the results of Haralampidis et al. [36] who investigated that the deletion of the STRE element from the *AtHsp90–1* promoter caused a reduction in the promoter activity under heat stress. CAAT-box and TATA-box act as binding sites for transcription factors. They are crucial in promoter activity, and they are almost universal in all PRs. The CAAT-box regulated expression of tissue-specific legumin gene in pea [37]. Although Zuo and Li [38] revealed that the TATA-box is not conserved in all plant genomes, it was conserved in all wheat PA biosynthesis promoters. MYB is also a main stress-responsive TFs and is extensively involved in gene expression regulation through controlling diverse biochemical pathways in plants under stress [39]. It is recognized that MYB TFs combine their relevant elements to regulate responses in the signaling, cellular, morphogenesis, and metabolic processes [40]. The Os*SAMDC* gene regulation was controlled by binding of MYB TFs to MYB *Cis*-elements in rice [41].

The present analyses revealed that more than 80% of the wheat PAs biosynthesis promoters contain single or multiple G-box, ABRE, as-1, TGACG-motif and CGTCA-motif CREs. The G-box (CACGTG) is a global CRE in plants, mediating gene expression through binding with G-box factors (GBFs) and is involved in light-responsiveness [42–44]. ABRE is another CRE that regulates ABA-responsiveness by binding to ABRE TF and activating dehydration-inducible gene expression [45, 46]. The *areb1*/*areb2*/*abf3* triple mutant has impaired dehydration-inducible gene expression and reduced dehydration stress tolerance [47]. The presence of STRE and AREB elements in PA biosynthesis gene promoters may explain the stimulation of endogenous PAs in wheat and different plant species by drought [48, 49] The element as-1 named (activation sequence-1), was first defined in viral and bacterial promoters but in plants, it is triggered in response to biotic stress [50] and it was found to be mediated by oxidative species [51]. TGACG- and CGTCA**-**motifs are engaged in MeJA responsiveness. Likely, they activate a series of defence processes in response to different abiotic stresses like drought and low temperature in plants [52].

Involvement of two hormone-responsive CREs (AREB and MeJA responsive elements) suggests presence of hormonal signaling pathway in the molecular base of PA in regulating functions in wheat. This may in part consistent with the results of Liu et al. [24]. in tomato as they found nine hormonal CREs. Therefore, analyses of *Cis*-elements in PA biosynthesis genes in wheat suggest that PAs regulate growth and response to drought through combining signals from hormonal and environmental factors, especially light responsive CREs.

### Regulation of PA biosynthesis during developmental stages in wheat

*In silico* gene expression analysis during wheat development showed that *TaSAMDC4*, *TaSAMDC5* and *TaSPDS* were the most expressed genes in all samples throughout all stages, which means that they are vital for wheat through its life stages. Contrarily, *TaACL5–2* expressed only at the reproductive stage, whereas *TaADC1,2* showed expression only in leaves and spike samples and *TaADC4* in roots only. Based on these expression data, it is possible that *TaADC4* plays a role in root development, whereas *TaADC1 and TaADC2* may play roles in spikes and leaves. Interestingly, the common expression of *TaSAMDC4*, *TaSAMDC5* and *TaSPDS* at all stages suggests that they do not have a definite role or tissue-specific function in wheat*.*

*In silico* analysis of gene expression in spikes showed that *TaSAMDC5* and *TaSAMDC6* expressed through the spike stages >8 TPM (transcript per million) relative expression. However, a few genes are expressed at certain spike developmental stages, such as *TaADC2* which is expressed only at the vegetative stage of spike formation. This suggests that *TaADC2* could play a role in the vegetative stage of spike development.

Gene expression *in silico* analysis during grain development of wheat indicated that PA biosynthesis genes are differentially expressed according to grain tissue and stage. Four members of *TaAIH* (*TaAIH1–4*) showed expression during grain developmental phases (milk, soft dough, and hard dough) but not in the final maturity stage. Notably, *TaAIH2* is expressed in the whole grain tissues except the starchy endosperm and aleurone layer. *TaADC1*, *TaADC2*, *TaADC3*, *TaODC2*, *TaODC3* and *TaSAMD6* are nearly not expressed in any grain developmental stage or any tissues, indicating that these genes do not function in grain development in wheat. Gong et al. [33] revealed that *ADC* and *ODC* are not important for fruit ripening. These findings with the present transcriptional data in the course of spike and grain development are in agreement in part with results of [53], who suggested that *ADC* is important for floral bud initiation whereas *ODC* is required for the subsequent stages of floral buds’ development.

Interestingly, gene expression analyses in the current study showed that some hoeomologs were differentially expressed and some homoeologs (*TaODC2*, *TaODC2* and *TaACL5–1*) were not expressed at any growth stage or expressed only at a certain stage or showed wide expression in the whole samples. This differential response of homoeologs is common in allopolyploids while subjected to stress. Ebeed et al. [54] investigated different expressions in the homoeologous wheat *PEX11* genes under ABA treatment and Dong and Adams [55] observed differential expression of homoeologous genes in allotetraploid cotton under different stresses. Investigations on polyploids have demonstrated that homoloci frequently contribute inequitably to levels of overall gene expression. For instance, they might be variably regulated under stress [56], or tissue-specific regulation [57]. Rapid and unique changes in gene expression, ranging from minor variations in the expression of homoloci to the full absence of expression caused by epigenetic homologs silencing, are a distinguishing characteristic of polyploid genomes [58]. The varied expression profiles of the PA biosynthesis homoeologs in the hexaploid wheat imply that they underwent transcriptional and functional differentiation during the development of wheat.

### Regulation of PA biosynthesis by drought in wheat

At 45 DAE, samples were collected from the root, shoot, spikes, and leaves and used to assess PA levels in tissues and the transcriptional profile of the biosynthesis genes.

The results of the quantification of PAs under normal conditions showed that Spm was not detected in roots whereas Spd was the highest PA in the root, shoot and spike tissues.Put and total PAS accumulated in wheat leaves more than in the other tissues. These results are in harmony with Takahashi et al. [11] who investigated the high concentration of Spd in roots, stems and inflorescence, whereas the higher Put levels were noticed in the *Brachypodium* leaves. Under drought stress, PAs distribution in tissues partially changed. A higher level of Spd was noticed in spikes and Put in leaves, whereas Spm was elevated in shoot and root tissues. This may point to the conversion of Spd to Spm in the shoot and root tissues by drought. This suggestion was elucidated by real-time PCR gene expression results that indicated downregulation of *TaSPDS* and upregulation of *TaSAMDC1–3* and *TaSPMS* in the roots to 2.5 and 10 FC, respectively. Drought likely upregulated *TaSPMS* and *TaSAMDC1–3* in the shoots to about 5 and 6 FC. However, the elevated levels of *TaSPDS* transcripts in shoot by drought without increment of Spd in shoot suggest the presence of post-transcriptional modification or oxidation of Spd by polyamine oxidase under stress conditions. Concomitantly with the result of transcriptional data [59], the importance of Spd and *TaSPDS* in the flowering stage was reflected.

Drought treatment reduced Put levels in the spikes and increased Put levels in the roots. According to the findings, *TaAIH* and *TaNLP1* transcripts may regulate Put biosynthesis during drought. The roots are the first organ to sense drought stress, but the induction of a particular gene expression depends on many factors such as the molecular network and the regularity mechanism. The regulatory system involved in root response to drought stress is complex and incorporates a wide range of TFs and regulatory proteins. Analysis of promoters of the *AIH-1* and *AIH-2* genes revealed the presence of a low number of stress responsive *Cis*-elements as STR (1), MBS (3) and DRE Core (3) and the absence of DRE1 which is involved in regulation of the gene by ABA and drought (Supplementary Table S6 and S7). The decline of both transcripts in the spikes by drought concurrent with the unchange in the expression of *ADC* and *ODC* transcripts suggests that ADC is the Put biosynthesis path under drought in the spike. Takahashi et al. [11] reported a higher correlation between the *ADC* expression levels and Put contents in *B. distachyon*. In roots, both transcripts were downregulated by drought, while the *TaODC* transcript was upregulated, indicating that Put biosynthesis in wheat roots occurs via the ODC-pathway under drought stress. Our previous research [10] revealed that the ADC-path is the functional path for Put synthesis under drought in wheat seedlings. The presence of *TaODC* and *TaADC* in wheat indicates that wheat could have two alternative routes that could be differentially activated depending on the different growth conditions to generate Put from l-arginine or ornithine. This supports the presence of tissue-specific regulation of PAs biosynthesis, particularly under drought. However, more research is needed to identify and explain the physiological function(s) of each route. Under normal or drought stress, TSpm was not detected in any examined tissue (its level may be lower than the sensitivity level of the HPLC) although the significant *ACL5* transcript level was particularly in shoot and leaf tissue (>2 FC). In contrast, *ACL5* was primarily expressed in *Brachypodium* roots. Orthologous *ACL5* genes are found in many plant species [60, 61] and TSpm is reported to be engaged in rice growth [62]. Thus, the current study provides the structural analysis of PA biosynthesis genes for the first time and offers insights into the regulation of PAs in wheat tissues throughout wheat growth under drought conditions.

## Materials and Methods

### Sequence retrieval and gene identification

*Arabidopsis thaliana* protein sequences and information were downloaded from the TAIR database (http://www.arabidopsis.org). Ornithine decarboxylase: ODC is absent from the Arabidopsis genome, so the rice ODC protein sequence was used to identify wheat ODC homologs. The retrieved sequences were used for TBLASTn analysis with an E-value threshold of <1e^−15^ and an identity of 50% as the threshold in the *Triticum aestivum* genome assembly by IWGSC in the JGI database, followed by the removal of redundant sequences. Using EXPASY [63], the conserved domain (CD) search tool at NCBI, SMART database and Pfam to confirm each predicted PA biosynthesis-related protein sequence as sharing a common domain with corresponding reference homologs. The protein sequences with identified incomplete domains at the N- or C-terminus have been excluded. Multiple sequence alignments for the PA biosynthesis-related genes were generated using ClustalOmega in EBI (https://www.ebi.ac.uk/Tools/msa/clustalo/). A phylogenetic tree was performed with the neighbor-joining algorithm in MEGA (version 11.0.11) [64].

### Structural analyses of wheat PA biosynthesis genes

The putative protein sequences of PA biosynthesis-related genes in wheat were analysed by EXPASY. Conserved motifs were scanned by the online MEME (MultipleExpectationMaximization for Motif Elicitation: http://meme.sdsc.edu/meme/meme.html) programme using the protein sequence of each homolog, with the following parameters: a maximum number of motifs = 20 and motif width set as 6–50 amino acids. For gene structure analysis, coding and non-coding sequences of each homolog were downloaded from the IWGSC database (http://www.wheatgenome.org/) and then exon–intron structures were identified by comparing the genomic and coding sequences [65].

### Analysis of protein subcellular localization

The subcellular location of each wheat protein was predicted using Plant-mPloc [66], CELLO v.2.5 [67], and WoLF PSORT. Prediction consensus was predicted based on the majority of result probabilities (Supplementary Table S5) and confirmed using TMHMM-2.0 to predict membrane-bound proteins [68] and Predotar [69] and TPpred 3.0 [70] to detect the putative N-terminal targeting sequences. The subcellular localization of the *Arabidopsis* proteins was compared according to SUBA (the subcellular localization database of *Arabidopsis* proteins [34].

### Analysis of promoter *Cis*-regulatory elements (CREs)

Each PA biosynthesis gene’s promoter sequence (2 kb upstream of the 5′UTR) was downloaded from the JGI database and submitted to PlantCare for CRE prediction [71]. The obtained *Cis*-elements (Supplementary Fig. 1) were compared with each other and discussed considering the available literature.

### RNA-seq data analysis of PA biosynthesis gene expression

Wheat transcriptome profiling data was retrieved from the Gene Expression Omnibus (GEO accession number: GSE83287) [59] to study the transcription of the identified genes in wheat early spike development. Eight samples were selected at four developmental stages (vegetative, elongation, glum primordium differentiation, and floret differentiation stage). The sample accession numbers are as follows: GSM2198202, GSM2198203, GSM2198204, GSM2198205, GSM2198210, GSM2198211, GSM2198212, and GSM2198213, two samples as a biological replicate for each treatment. Values for the selected samples were used to compute the average of the read-counts in duplicates. *In-silico* analysis was done by using Genevestigator [72], gene expression atlas, EMBL-EBI, and Wheat Expression Browser (http://www.wheat-expression.com/).

### Plant growth experiment

To investigate PAs biosynthesis in different tissues, wheat (*T. aestivum* L. cv. Sakha 94) plants were planted in pots with soil (compost: vermiculite = 2:1) and cultivated one plant per pot in a growth chamber at 300 μmol/m^2^/s, 16 h/8 h (light/dark) at 25 °C, and relative humidity of 60–70%. Water stress was applied at 14 days after emergence (DAE) by withholding water. The roots, shoot axis, fifth leaves, and spikelets were harvested from the adult plants 45 at DAE. Samplings were repeated three times. Each sample was immediately frozen in liquid nitrogen and stored at −80 °C until use.

### Extraction and measurement of PAs by HPLC

PAs were extracted from 0.5 g plant samples and homogenized with ten volumes of 5% (v/v) cold perchloric acid. The mixtures were kept on ice for 1 h. Following centrifugation at 15,000 × g for 30 min at 4 °C, the supernatants were collected. Benzoyl chloride (10 μl) was added to 1 ml of plant extract and 1 ml of 2 N NaOH, then vortexed for 10 sec. Saturated sodium chloride (2 ml) and diethyl ether (2 ml) were added and mixed vigorously, and then, centrifuged at 3000 × g for 10 min at room temperature. PAs were analysed by HPLC at 254 nm, a flow rate of 1 ml/min. For separation and column washing, 42% acetonitrile was used.

### Total RNA Extraction and Quantitative Real-Time Polymerase Chain Reaction (qPCR) Analysis

About 0.05 g of tissue was used for total RNA isolation by Triazole (Bioline), as explained in the manufacturer’s procedure. Then, DNase I (Thermo Scientific) was applied to purify RNA samples. Synthesis of cDNA was done using 1 μg total RNA with a Sensifast 1st cDNA synthesis kit (Bioline). Primers were designed according to identified sequences of wheat PAs orthologous genes and are listed in Supplementary Table S8. A quantitative real-time polymerase chain reaction (qPCR) was performed with the diluted cDNA samples in a 20 μl reaction mixture containing 10 μl SensiFast SYBR Lo-Rox 2X mix (Bioline) and 1.2 μl (300n mole) of each primer (Supplementary Table S9). PCR was performed as follows: denaturation for 2 min at 95 °C, followed by 40 cycles of [10 s at 95 °C, 30 s at 56–60 °C] using a STRATAGENE MxPro-3000P. Relative expression was calculated using the 2-DDCt method, where the relative mRNA level was normalised against *GAPDH* (the internal standard gene) and compared with the control.

### Statistical analysis

Analysis of variance (ANOVA) was performed to identify significant differences between them at *P* ≤ 0.05. A comparison was made to determine the significant effects between the treatments, using the least significant difference (LSD) test with a *P* ≤ 0.05.

## Conclusions

In conclusion, the current study provides the structural analysis of PA biosynthesis genes for the first time and furthermore investigates their organ-specific expression profile using the publicly available RNAseq data and validates the results using quantitative real-time PCR. The analysis revealed 30 gene models were identified as putative genes responsible for PA biosynthesis in wheat. Two new motifs have been discovered by the analysis of protein sequences which were conserved in *AIH*, *SAMDC* and *SPMS* genes and they may be novel motifs for these proteins. The CREs distribution and conserveness of hormone and stress responsive elements in promoters reflect their functions in response to stress. Transcriptional analyses of the PA biosynthesis homologs in the hexaploid wheat suggest tissue-specific regulation particularly under drought stress and imply that they underwent transcriptional and functional differentiation during the wheat evolution. Overall, the current results shape our understanding of the functional characterization of biosynthesis pathway of Put, Spd and Spd in wheat and offers insights into the regulation of PAs in wheat tissues throughout wheat growth under drought conditions. Eventually, present a reference for the further functional examination of TaPA biosynthesis proteins for effective and consistent plant cultivation.

## Supplementary Information

**Additional file 1: Fig. S1.** Cis-elements obtained from PlantCare database for the promoter sequences of PA biosynthesis genes in wheat.

**Additional file 2: Table S1.** Blast results for PAs biosynthesis genes using *Arabidopsis thalisna* genes as queries.

**Additional file 3: Table S2.** Domain identification using CDD-NCBI search tool.

**Additional file 4: Table S3.** SMART analysis for PAs biosynthesis related gene homologs in wheat.

**Additional file 5: Table S4.** Functional domains identified by Pfam in the putative PAs biosynthesis gene homologs in wheat.

**Additional file 6: Table S5.** The significant prediction of subcellular location for Arabidopsis and wheat PA biosynthesis proteins.

**Additional file 7: Table S6.** Distribution of Cis-elements of the promoter regions of PAs biosynthesis genes in wheat.

**Additional file 8: Table S7.** Summary of Cis-elements number and % in the promoter regions of PAs biosynthesis genes in wheat.

**Additional file 9: Table S8.** The Gene expression values by high throughput sequencing of spike tissue at different stages.

**Additional file 10: Table S9.** Primer sequences used in the study for wheat PAs genes.

## Data Availability

The data and datasets used and/or analyzed in the current study are available from the following repositories: TAIR (https://www.arabidopsis.org/), Phytozome (https://phytozome-next.jgi.doe.gov/) and Gene Expression Omnibus (https://www.ncbi.nlm.nih.gov/geo/). The original contributions presented in the study are included in the article and its supplementary information files.

## Abbreviations

PAs: Polyamines
Put: Putrescine
Spd: Spermidine
Spm: Spermine
Tspm: Thermospermine
Cad: Cadaverine
Arg: Arginine
ACL5: Thermospermine synthase
ADC: Arginine decarboxylase
AIH: agmatine iminohydrolase
ODC: Ornithine decarboxylase
NLP1: N-carbamoylputrescine amidohydrolase/Nitrlase like protein 1
SAMDC: S-adenosylmethionine decarboxylase
SPDS: Spermidine synthase
SPMS: Spermine synthase
BLAST: Basic local alignment search tool
CREs: *Cis*-acting elements
STRE: Stress-responsive element
ABRE: Abscisic acid-responsive element
