# Supplementary figures and images for: Genome-wide analysis of polyamine biosynthesis genes in wheat reveals gene expression specificity and involvement of STRE and MYB-elements in regulating polyamines under drought
Source: BMC Genomics. 2022 Oct 30;23:734. doi: 10.1186/s12864-022-08946-2 (PMC9618216; doi:10.1186/s12864-022-08946-2)

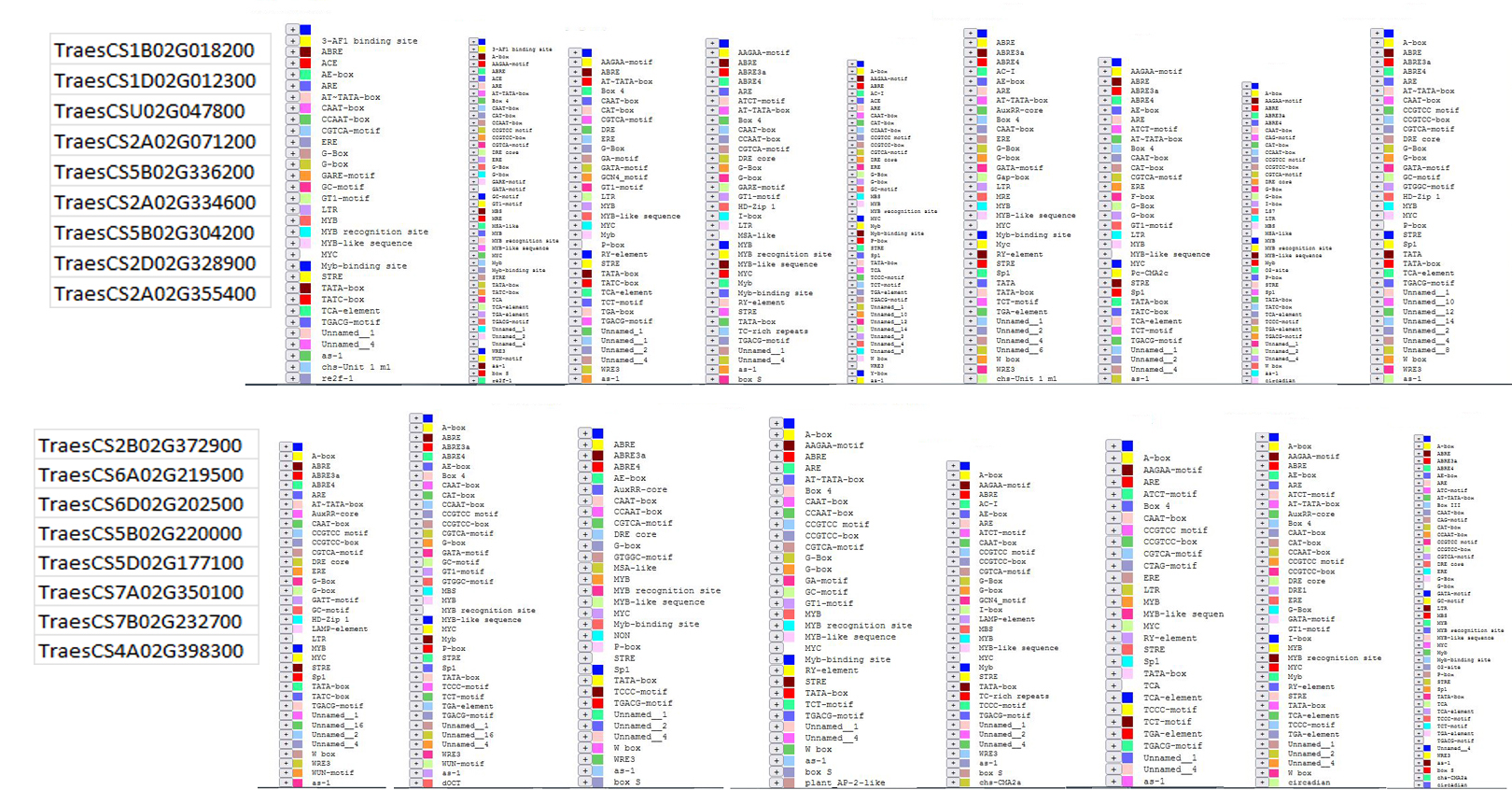

Supplement: Supplementary file 1 — Additional file 1: Fig. S1. Cis-elements obtained from PlantCare database for the promoter sequences of PA biosynthesis genes in wheat. [file 12864_2022_8946_MOESM1_ESM.jpg]
