# Supplementary material for: Genome-wide analysis of polyamine biosynthesis genes in wheat reveals gene expression specificity and involvement of STRE and MYB-elements in regulating polyamines under drought
Source: BMC Genomics. 2022 Oct 30;23:734. doi: 10.1186/s12864-022-08946-2 (PMC9618216; doi:10.1186/s12864-022-08946-2)
Supplement: Supplementary file 6 — Additional file 6: Table S5. The significant prediction of subcellular location for Arabidopsis and wheat PA biosynthesis proteins. [file 12864_2022_8946_MOESM6_ESM.docx]

**Supplementary Table S5**. The significant prediction of subcellular location for Arabidopsis and wheat PA biosynthesis proteins.

| **Gene Name** | **Wheat gene** | **Plant-mPloc** | **Cello life** | **Psort** | **TPpred 3.0** |
| --- | --- | --- | --- | --- | --- |
| Arginine decarboxylase | *TaADC1* | Chloroplast. Cytoplasm. Peroxisome | Chloroplast. Cytoplasm. Peroxisome | Cytoplasm | cytoplasm |
|  | *TaADC2* | Chloroplast. Cytoplasm. | Chloroplast. Cytoplasm. | NP | cytoplasm |
|  | *TaADC3* | Chloroplast | Chloroplast | Cytoplasm | cytoplasm |
|  | *TaADC4* | Chloroplast | Chloroplast | Mitochondria, Chloroplast | nucleus |
| Ornithine decarboxylase | *TaODC1* | Chloroplast | Chloroplast | Extra | cytoplasm |
|  | *TaODC2* | Chloroplast | Chloroplast | Extra | cytoplasm |
|  | *TaODC3* | Chloroplast | Chloroplast | Extra | cytoplasm |
| Agmatine iminohydrolase | *TaAIH1* | Chloroplast | Chloroplast | Cytoplasm | cytoplasm |
|  | *TaAIH2* | Chloroplast | Chloroplast | Cytoplasm | cytoplasm |
|  | *TaAIH3* | Chloroplast | Chloroplast | Cytoplasm | cytoplasm |
|  | *TaAIH4* | Chloroplast. Nucleus. | Chloroplast. Nucleus. | Cytoplasm | cytoplasm |
|  | *TaAIH5* | Chloroplast | Chloroplast | Mitochondria | mitochondrion |
| N-carbamoyl putrescine amidohydrolase | *TaNLP1-1* | Cell membrane | Cell membrane | Cytoplasm | cytoplasm |
|  | *TaNLP1-2* | Cell membrane | Cell membrane | Cytoplasm | cytoplasm |
| S-adenosylmethionine decarboxylase | *TaSAMDC1* | Cell membrane. Nucleus | Cell membrane. Nucleus | Nucleus, Chloroplast | nucleus |
|  | *TaSAMDC2* | Cell membrane. Chloroplast. Nucleus | Cell membrane. Chloroplast. Nucleus | Cytoskeleton | nucleus |
|  | *TaSAMDC3* | Cell membrane. Chloroplast. Nucleus | Cell membrane. Chloroplast. Nucleus | Cytoskeleton | nucleus |
|  | *TaSAMDC4* | Cell membrane. Chloroplast. Nucleus | Cell membrane. Chloroplast. Nucleus | Cytoskeleton | nucleus |
|  | *TaSAMDC5* | Cell membrane. Chloroplast. Nucleus | Cell membrane. Chloroplast. Nucleus | Cytoskeleton | nucleus |
|  | *TaSAMDC6* | Cell membrane. Nucleus. | Cell membrane. Nucleus. | Nucleus | nucleus |
|  | *TaSAMDC7* | Chloroplast | Chloroplast | Nucleus | nucleus |
| Spermidine synthase | *TaSPDS* | Cytoplasm | Cytoplasm | Chloroplast | cytoplasm |
| Spermine synthase | *TaSPMS1* | Chloroplast. Cytoplasm. | Chloroplast. Cytoplasm. | cytoskeleton | cytoplasm |
|  | *TaSPMS2* | Cytoplasm | Cytoplasm | cytoskeleton | cytoplasm |
|  | *TaSPMS3* | Cytoplasm | Cytoplasm | cytoplasm | cytoplasm |
|  | *TaSPMS4* | NP | NP | chloroplast | chloroplast |
|  | *TaSPMS5* | Cytoplasm | Cytoplasm | cytoplasm | cytoplasm |
|  | *TaSPMS6* | Cytoplasm | Cytoplasm | cytoplasm | cytoplasm |
| ACAULIS 5 | *TaACL5-1* | Chloroplast. Cytoplasm. | Chloroplast. Cytoplasm. | Peroxisomes | cytoplasm |
|  | *TaACL5-2* | Chloroplast | Chloroplast | Mitochondria | cytoplasm |

^NP: not predicted^
