# Supplementary material for: Genome-wide analysis of polyamine biosynthesis genes in wheat reveals gene expression specificity and involvement of STRE and MYB-elements in regulating polyamines under drought
Source: BMC Genomics. 2022 Oct 30;23:734. doi: 10.1186/s12864-022-08946-2 (PMC9618216; doi:10.1186/s12864-022-08946-2)
Supplement: Supplementary file 7 — Additional file 7: Table S6. Distribution of Cis-elements of the promoter regions of PAs biosynthesis genes in wheat. [file 12864_2022_8946_MOESM7_ESM.docx]

**Supplementary Table 6**. Distribution of *Cis*-elements of the promoter regions of PAs biosynthesis genes in wheat.

| ***Cis*-elements** | | | **PAs biosynthesis genes** | | | | | | | | | | | | | | | | | | | | |
| --- | --- | --- | --- | --- | --- | --- | --- | --- | --- | --- | --- | --- | --- | --- | --- | --- | --- | --- | --- | --- | --- | --- | --- |
|  |  |  | **ADC** | | | | **ODC** | | **AIH** | | | **SAMDC** | | | | | | **SPMS** | | | | **ACL5** | |
| **Category of *Cis*-elements** | **Name** | **Function** | TraesCS1B02G018200 | TraesCS1D02G012300 | TraesCSU02G047800 | TraesCS2A02G071200 | TraesCS5B02G336200 | TraesCS5B02G304200 | TraesCS2A02G334600 | TraesCS2D02G328900 | TraesCS2A02G355400 | | TraesCS2B02G372900 | TraesCS6A02G219500 | TraesCS6D02G202500 | TraesCS5B02G220000 | TraesCS5D02G177100 | | TraesCS7A02G350100 | TraesCS7B02G232700 | TraesCS4A02G398300 | |  |
| Light responsive elements | 4cl-CMA1b | light responsive element |  |  |  |  |  |  |  |  |  | |  |  |  |  |  | |  |  | 0 | |  |
|  | 3-AF1 binding site | light responsive element |  | 1 |  |  |  |  |  |  |  | |  |  |  |  |  | |  |  |  | |  |
|  | ATC-motif | part of a cons. DNA module involved in light responsiveness |  |  |  |  |  |  |  |  |  | |  |  |  |  |  | |  |  | 1 | |  |
|  | ATCT-motif | cis-acting regulatory element associated to TGAGTCA motif |  |  |  | 1 |  |  | 1 |  |  | |  |  |  |  | 1 | | 1 | 1 | 2 | |  |
|  | ACE | cis-acting element involved in light responsiveness | 1 | 1 |  |  | 1 |  |  |  |  | |  |  |  |  |  | |  |  |  | |  |
|  | AE-box | part of a module for light response | 1 |  |  |  |  | 1 | 2 |  |  | |  | 1 | 1 |  | 1 | |  | 1 | 1 | |  |
|  | Box4 | part of a cons. DNA module involved in light responsiveness |  | 1 | 1 | 1 |  | 4 | 2 |  |  | |  | 1 |  | 1 |  | | 1 | 1 |  | |  |
|  | CG-motif | part of a light responsive element |  |  |  |  |  |  |  |  | 2 | |  |  |  |  |  | |  |  |  | |  |
|  | chs-CMA2a | part of a light responsive element |  |  |  |  |  |  |  |  |  | |  |  |  |  | 1 | |  |  | 1 | |  |
|  | chs-Unit 1 m1 | part of a light responsive element | 1 |  |  |  |  | 1 |  |  |  | |  |  |  |  |  | |  |  |  | |  |
|  | CAG-motif | part of a light responsive element |  |  |  |  |  |  |  | 1 |  | |  |  |  |  |  | |  |  | 1 | |  |
|  | GA-motif | part of a light responsive element |  |  | 1 |  |  |  |  |  |  | |  |  |  | 1 |  | |  |  |  | |  |
|  | Gap-box | part of a light responsive element |  |  |  |  |  | 2 |  |  |  | |  |  |  |  |  | |  |  |  | |  |
|  | GATA-motif | part of a light responsive element |  | 1 | 1 |  |  | 1 |  |  | 1 | |  | 2 |  |  |  | |  | 1 | 2 | |  |
|  | GATT-motif | part of a light responsive element |  |  |  |  |  |  |  |  |  | | 1 |  |  |  |  | |  |  |  | |  |
|  | G-Box | cis-acting regulatory element involved in light responsiveness | 3 | 2 | 2 | 1 | 1 | 1 | 1 | 1 | 3 | | 2 |  |  | 3 | 2 | |  | 2 | 1 | |  |
|  | G-box | cis-acting regulatory element involved in light responsiveness | 5 | 5 |  | 4 | 3 | 5 | 5 | 2 | 5 | | 4 | 4 | 4 | 4 | 5 | |  |  | 4 | |  |
|  | GT1-motif | light responsive element | 2 | 1 | 3 | 2 |  |  | 1 |  |  | |  | 1 |  | 3 |  | |  | 1 |  | |  |
|  | GTGGC-motif | part of a light responsive element |  |  |  |  |  |  |  |  | 1 | |  | 2 | 1 |  |  | |  |  |  | |  |
|  | I-box | part of a light responsive element |  |  |  | 1 |  |  |  | 1 |  | |  |  |  |  | 1 | |  | 2 |  | |  |
|  | LAMP-element | part of a light responsive element |  |  |  |  |  |  |  |  |  | | 1 |  |  |  | 1 | |  |  |  | |  |
|  | MRE | MYB binding site involved in light responsiveness |  | 2 |  |  |  | 3 |  |  |  | |  |  |  |  |  | |  |  |  | |  |
|  | Sp1 | light responsive element |  |  |  |  | 2 | 2 | 1 | 1 | 1 | | 1 | 2 | 1 |  |  | | 1 |  | 1 | |  |
|  | TCCC-motif | part of a light responsive element |  |  |  |  | 1 |  |  | 1 |  | |  | 1 | 1 |  | 1 | | 1 | 2 | 1 | |  |
|  | TCT-motif | part of a light responsive element |  |  | 2 |  | 1 | 3 | 2 |  |  | |  | 1 |  | 2 |  | | 3 |  |  | |  |
|  | Pc-CMA2c | part of a light responsive element |  |  |  |  |  |  | 1 |  |  | |  |  |  |  |  | |  |  |  | |  |
|  | LS7 | part of a light responsive element |  |  |  |  |  |  |  | 1 |  | |  |  |  |  |  | |  |  |  | |  |
|  | Box II | part of a light responsive element |  |  |  |  |  |  |  |  |  | |  |  |  |  |  | |  |  |  | |  |
| Hormone responsive elements | ABRE | cis-acting element involved in the ABA responsiveness | 5 | 4 | 3 | 5 | 3 | 3 | 4 | 5 | 7 | | 7 | 4 | 3 | 4 | 7 | |  | 1 | 3 | |  |
|  | ABRE3a | cis-acting element involved in the ABA responsiveness |  |  |  | 1 |  | 1 | 1 | 1 | 1 | | 1 | 2 | 1 |  |  | |  |  | 1 | |  |
|  | ABRE4 | cis-acting element involved in the ABA responsiveness |  |  |  | 1 |  | 1 | 4 | 1 | 1 | | 1 | 2 | 1 |  |  | |  |  | 1 | |  |
|  | ERE | ethylene-responsive element | 2 | 1 | 3 |  | 1 | 1 | 1 |  |  | | 1 |  |  |  |  | | 1 | 2 | 1 | |  |
|  | TGACG-motif | cis-acting regulatory element involved in the MeJA-responsiveness | 2 | 4 | 5 | 4 | 2 |  | 2 | 1 | 1 | | 1 | 2 | 1 | 2 | 4 | | 1 |  | 1 | |  |
|  | AuxRR-core | cis-acting regulatory element involved in Aux responsiveness |  |  |  |  |  | 1 |  |  |  | | 1 |  | 1 |  |  | |  | 1 |  | |  |
|  | GARE-motif | gibberellin-responsive element | 2 | 1 |  | 1 |  |  |  |  |  | |  |  |  |  |  | |  |  |  | |  |
|  | P-box | gibberellin-responsive element |  |  | 1 |  | 1 |  |  | 2 | 1 | |  | 1 | 2 |  |  | |  |  | 1 | |  |
|  | TATC-box | cis-acting regulatory element involved in the MeJA-responsiveness | 1 | 1 | 1 |  |  |  | 1 | 2 |  | | 1 |  |  |  |  | |  |  |  | |  |
|  | TCA-element | cis-acting element involved in salicylic acid responsiveness | 1 | 1 | 2 |  |  |  | 1 | 1 | 1 | |  |  |  |  |  | | 1 | 1 | 1 | |  |
|  | TCA | cis-acting element involved in salicylic acid responsiveness |  | 1 |  |  | 2 |  |  |  |  | |  |  |  |  |  | | 1 |  | 2 | |  |
|  | TGA-box | part of an auxin-responsive element |  |  | 1 |  | 4 |  |  |  |  | |  |  |  |  |  | |  |  | 1 | |  |
|  | TGA-element | auxin-responsive element |  | 1 |  |  | 1 | 1 |  | 1 |  | |  | 1 |  |  |  | | 1 | 2 |  | |  |
|  | CGTCA-motif | cis-acting regulatory element involved in the MeJA-responsiveness | 1 | 4 | 5 | 4 | 2 |  | 2 | 1 | 1 | | 1 | 2 | 1 | 2 | 4 | | 1 |  | 1 | |  |
| Environmental stress-related element | LTR | cis-acting element involved in low-temperature responsiveness | 1 |  | 1 | 1 |  | 1 | 1 | 2 |  | | 3 |  |  |  |  | | 1 |  | 1 | |  |
|  | MBS | MYB binding site involved in drought-inducibility |  | 1 |  |  | 2 |  |  | 3 |  | |  | 1 |  |  | 2 | |  |  | 2 | |  |
|  | TC-rich repeats | cis-acting element involved in defense and stress responsiveness |  |  |  | 1 |  |  |  |  |  | |  |  |  |  | 1 | |  |  |  | |  |
|  | ARE | cis-acting regulatory element essential for the anaerobic induction | 5 | 5 |  | 1 | 1 | 2 | 2 |  | 1 | | 1 |  |  | 1 | 2 | | 1 | 2 | 1 | |  |
|  | box S | elicitation,wounding and pathogen responsievness |  | 1 |  | 1 |  |  |  |  |  | |  |  | 1 | 2 | 2 | |  |  | 3 | |  |
|  | DRE Core | cis-acting element involved in dehydration, low-temp, salt stresses |  | 1 | 1 | 1 | 2 |  |  | 3 | 1 | | 1 |  | 1 |  |  | |  | 1 | 1 | |  |
|  | DRE1 | cis-elements are involved in regulation of the gene by ABA and drought |  |  |  |  |  |  |  |  |  | |  |  |  |  |  | |  | 1 |  | |  |
|  | W box | wounding and pathogen respons |  |  |  |  | 1 | 2 |  | 3 | 3 | | 1 |  | 1 | 4 |  | |  | 2 |  | |  |
|  | WUN-motif | wound-responsive element |  | 1 |  |  |  |  |  |  |  | | 1 | 1 |  |  |  | |  |  |  | |  |
|  | WRE3 | wound-responsive element |  | 1 | 1 |  | 2 | 1 |  |  | 2 | | 3 | 3 | 2 |  | 4 | |  |  | 2 | |  |
|  | STRE | stress responsive element | 8 | 6 | 1 | 3 | 2 | 2 | 3 | 1 | 3 | | 2 | 3 | 3 | 6 | 4 | | 3 | 4 | 2 | |  |
|  | GC-motif | enhancer-like element involved in anoxic specific inducibility | 1 | 1 |  |  | 2 |  |  |  |  | | 3 | 1 |  | 1 |  | |  |  | 1 | |  |
| Devolepment related elements | AAGAA-motif | involved in endosperm-specific negative expression |  | 1 | 2 | 3 | 2 |  | 2 | 2 |  | |  |  |  | 1 | 3 | | 1 | 1 |  | |  |
|  | AC-I | element involved in negative regulation on phloem expression; and responsible for restricting the vascular expression to the xylem |  |  |  |  | 1 | 1 |  |  |  | |  |  |  |  | 1 | |  |  |  | |  |
|  | AC-II | element involved in negative regulation of phloem expression; and responsible for restricting the vascular expression to the xylem |  |  |  |  |  |  |  |  |  | |  |  |  |  |  | |  |  |  | |  |
|  | O2-site | cis-acting regulatory element involved in zein metabolism regulation |  |  |  |  |  |  |  | 2 |  | |  |  |  |  |  | |  |  | 3 | |  |
|  | as1 | cis-acting regulatory element involved in the root-specific expression |  | 4 | 5 | 4 | 2 |  | 2 | 1 | 1 | | 1 | 2 | 1 | 2 | 4 | | 1 |  | 1 | |  |
|  | CAT-box | cis-acting regulatory element related to meristem expression |  | 1 | 1 |  | 2 |  | 1 | 2 |  | |  | 1 |  |  |  | |  | 1 | 1 | |  |
|  | CCAAT-box | cis-acting regulatory element related to meristem expression | 1 | 1 |  | 1 | 3 |  |  | 1 |  | |  | 1 | 3 |  |  | |  | 1 | 2 | |  |
|  | circadian | cis-acting regulatory element involved in circadian control |  |  |  |  |  |  |  | 1 |  | |  |  |  |  |  | |  | 1 | 1 | |  |
|  | GCN4_motif | cis-regulatory element involved in endosperm expression |  |  | 1 |  |  |  |  |  |  | |  |  |  |  | 3 | |  |  |  | |  |
|  | RY-element | cis-acting regulatory element involved in seed-specific regulation |  |  | 1 | 1 |  | 1 |  |  |  | |  |  |  | 1 |  | | 1 | 1 |  | |  |
|  | HD-Zip-1 | element involved in differentiation of the palisade mesophyll cells |  |  |  | 2 |  |  |  |  | 1 | | 1 |  |  |  |  | |  |  |  | |  |
|  | MSA-like | cis-acting element involved in cell cycle regulation |  | 2 |  | 1 |  |  |  | 1 |  | |  |  | 1 |  |  | |  |  |  | |  |
|  | CCGTCC-box | development related motifs involved in activation of meristem specific expression |  | 3 |  |  | 4 |  |  | 1 | 2 | | 2 | 1 |  | 1 | 1 | | 6 | 1 | 1 | |  |
| Promoter related element | A-box | cis-acting regulatory element |  | 3 |  |  |  |  |  | 1 | 2 | | 2 | 1 |  | 1 | 1 | | 6 | 1 | 1 | |  |
|  | CAAT-box | common cis-acting element in promoter and enhancer regions | 26 | 25 | 28 | 31 | 27 | 30 | 25 | 14 | 18 | | 20 | 22 | 5 | 33 | 34 | | 18 | 25 | 31 | |  |
|  | CCGTCC-motif | cis-acting regulatory element related to meristem specific activation |  | 3 |  |  | 4 |  |  | 1 | 2 | | 2 | 1 |  | 1 | 1 | | 6 | 1 | 1 | |  |
|  | TATA-box | core promoter element around -30 of transcription start | 18 | 15 | 46 | 16 | 6 | 16 | 13 | 3 | 17 | | 23 | 8 | 2 | 38 | 15 | | 23 | 33 | 6 | |  |
|  | TATA-box (TATATA) | TATA-like hexamers | 2 | 2 | 6 | 1 |  | 2 | 1 |  | 3 | | 1 |  |  | 1 |  | |  | 1 | 1 | |  |
|  | MYB | Transcription factor-CAACAG | 7 | 7 | 4 | 5 | 3 | 7 | 3 | 2 | 1 | | 1 | 1 | 5 | 5 | 5 | | 2 | 1 | 4 | |  |
|  | Myb | Transcription factor-CAACTG |  | 1 | 1 | 1 | 2 |  |  | 4 |  | |  | 1 |  |  | 2 | |  | 1 | 2 | |  |
|  | MYB-like sequence | MYB-like sequence | 2 | 1 | 2 | 2 |  | 2 | 3 | 1 |  | |  | 1 | 1 | 1 | 3 | | 2 |  | 1 | |  |
|  | MYC | Transcription factor | 6 | 4 | 6 | 4 | 8 | 6 | 2 |  | 5 | | 2 | 4 | 1 | 9 | 2 | | 7 | 6 | 3 | |  |
|  | Myc | Transcription factor |  |  |  |  |  | 1 |  |  |  | |  |  |  |  |  | |  |  |  | |  |
| Site-binding related elements | Unnamed_1 | 60K protein binding site | 5 | 5 | 2 | 5 | 3 | 2 | 4 | 2 | 9 | | 9 | 5 | 2 | 2 | 3 | | 4 | 2 |  | |  |
|  | 3-AF1 | 3-AF1 binding site | 1 |  |  |  |  |  |  |  |  | |  |  |  |  |  | |  |  |  | |  |
|  | Unnamed_6 | SEF4 factor binding site |  |  |  |  |  | 1 |  |  |  | |  |  |  |  |  | |  |  |  | |  |
|  | Myb-binding site | Myb-binding site | 2 | 3 |  | 2 | 2 | 1 |  |  |  | |  |  | 1 | 1 |  | |  |  | 1 | |  |
|  | MYB recognition site | MYB recognition site | 1 | 1 |  | 1 | 3 |  |  | 1 |  | |  | 1 | 3 | 1 |  | |  | 1 | 2 | |  |
|  | CCAAT-box | MYBHv1 binding site |  |  |  |  |  |  |  |  |  | | 1 |  |  | 1 |  | |  |  |  | |  |
|  | Box III | protein binding site |  |  |  |  |  |  |  |  |  | |  |  |  |  |  | |  |  | 1 | |  |
| Other elements | CTAG-motif | unkown |  |  |  |  |  |  |  |  |  | |  |  |  |  |  | | 1 |  |  | |  |
|  | F-box | unkown |  |  |  |  |  |  | 1 |  |  | |  |  |  |  |  | |  |  |  | |  |
|  | re2f-1 | unknown | 1 | 1 |  |  |  |  |  |  |  | |  |  |  |  |  | |  |  |  | |  |
|  | plant_AP-2-like | unknown |  |  |  |  |  |  |  |  |  | |  |  |  | 1 |  | |  |  |  | |  |
|  | Unnamed_2 | unkown |  | 1 | 2 |  | 1 | 1 | 2 | 1 | 4 | | 3 |  | 1 |  | 1 | |  | 1 |  | |  |
|  | Unnamed_4 | unkown | 27 | 29 | 1 | 12 | 33 | 20 | 11 | 23 | 26 | | 18 | 20 | 26 | 16 | 16 | | 14 | 14 | 33 | |  |
|  | Unnamed_8 | unkown |  |  |  |  | 1 |  |  |  | 1 | |  |  |  |  |  | |  |  |  | |  |
|  | Unnamed_10 | unkown |  |  |  |  | 1 |  |  |  | 1 | |  |  |  |  |  | |  |  |  | |  |
|  | Unnamed_12 | unkown |  |  |  |  | 1 |  |  |  | 1 | |  |  |  |  |  | |  |  |  | |  |
|  | Unnamed_14 | unkown |  |  |  |  | 1 |  |  |  | 1 | |  |  |  |  |  | |  |  |  | |  |
|  | Unnamed_16 | unknown |  |  |  |  |  |  |  |  |  | |  | 1 |  |  |  | |  |  |  | |  |
|  | Unnamed_16 | unknown |  |  |  |  |  |  |  |  |  | | 1 |  |  |  |  | |  |  |  | |  |
|  | Y-box | unknown |  |  |  |  | 1 |  |  |  |  | |  |  |  |  |  | |  |  |  | |  |
|  | dOCT | unknown |  |  |  |  |  |  |  |  |  | |  | 1 |  |  |  | |  |  |  | |  |
|  | NON | unknown |  |  |  |  |  |  |  |  |  | |  |  | 1 |  |  | |  |  |  | |  |
|  | Unnamed_5 | unknown |  |  |  |  |  |  |  |  |  | |  |  |  |  |  | |  |  |  | |  |
