# Supplementary material for: Genome-wide analysis of polyamine biosynthesis genes in wheat reveals gene expression specificity and involvement of STRE and MYB-elements in regulating polyamines under drought
Source: BMC Genomics. 2022 Oct 30;23:734. doi: 10.1186/s12864-022-08946-2 (PMC9618216; doi:10.1186/s12864-022-08946-2)
Supplement: Supplementary file 10 — Additional file 10: Table S9. Primer sequences used in the study for wheat PAs genes. [file 12864_2022_8946_MOESM10_ESM.docx]

**Table S9: Primer sequences used in the study for wheat PAs genes**

| **Name** | **Primer pairs** | **Sequence (5'->3')** | **Length** | **Tm** | **GC%** | **Product size** |
| --- | --- | --- | --- | --- | --- | --- |
| ***TaADC***  TraesCS1D02G012300 TraesCS1B02G018200 | Fw | 5'- CAACGACTTTGTTAGCTTTGG -3' | 21 | 56.02 | 42.86 | 104 |
|  | Rv | 5'- CAGGCTTGGCTTTGGTAA-3' | 18 | 55.1 | 50 |  |
| ***TaODC3***  TraesCS5B02G336200 | Fw | 5'- GGCCACTTCTTCTAGGTTCA-3' | 20 | 56.85 | 50 | 76 |
|  | Rv | 5'- ACTCGGCGTCTTATATAGCG-3' | 20 | 57.1 | 50 |  |
| ***TaSAMDC1-3***  TraesCS6A02G219500  TraesCS6D02G202500  TraesCS6B02G249000 | Fw | 5'- CGAGCTTGTGTTGCGTCAG-3' | 19 | 59.8 | 57.89 | 117 |
|  | Rv | 5'- ATACATTCGCTCACACTGGCA-3' | 21 | 60.07 | 47.62 |  |
| ***TaSPMS***  TraesCS7B02G232700  TraesCS7A02G350100  TraesCS7D02G328500 | Fw | 5'- AGTAGAGAAGATTTTGTACCAGG-3' | 23 | 55.42 | 39.13 | 80 |
|  | Rv | 5'- GGACATTCCCATAGGTTGAAG-3' | 21 | 56.01 | 47.62 |  |
| ***TaSPDS***  TraesCS7B02G163500 | Fw | 5'- TCTGCTGTATGTCTCAGCTTGT-3' | 22 | 59.44 | 45.45 | 86 |
|  | Rv | 5'- AAATCCGAACGAACTGCCAT-3' | 20 | 58.18 | 45 |  |
| ***TaACL5-2*** TraesCS4A02G398300 | Fw | 5'- GTCCTCTTCTCGCTCGGCAA-3' | 20 | 62.21 | 60 | 106 |
|  | Rv | 5'- GGTGTCGTCGTACAAATTCTGC-3' | 22 | 60.16 | 50 |  |
| ***TaSAMDC4*** TraesCS2A02G355400 | Fw | 5'- ACCAATCTCTCTGGCTTGCTG-3' | 21 | 60.34 | 52.38 | 54 |
|  | Rv | 5'- GAAAGACTAGAGAACTGGTCGAT-3' | 23 | 57.43 | 43.48 |  |
| ***TaAIH***  TraesCS2D02G328900  TraesCS2A02G334600  TraesCS2B02G347800 | Fw | 5'- ATACATTCGCTCACACTGGCA-3' | 21 | 60.07 | 47.62 | 120 |
|  | Rv | 5'- TCTGCTGTATGTCTCAGCTTGT-3' | 22 | 59.44 | 45.45 |  |
| ***TaNLP1***  TraesCS5B02G022300  TraesCS5A02G024500 | Fw | 5'- CAGGCTTGGCTTTGGTAA-3' | 18 | 55.1 | 50 | 100 |
|  | Rv | 5'- GGCCACTTCTTCTAGGTTCA-3' | 20 | 56.85 | 50 |  |
| Wheat ref gene  **GAPDH** | Fw | 5'-TTGCTCTGAACGACCATTTC-3' | 20 | 60.7 | 45 | 175 |
|  | Rv | 5'-GACACCATCCACATTTATTCTTC-3' | 23 | 59.8 | 39.1 |  |
